# Phosphorylation of UDP-glucose dehydrogenase increases glycosaminoglycan biosynthesis and promotes tumor cell motility, spheroid growth, and therapeutic resistance

**DOI:** 10.1016/j.matbio.2025.10.004

**Published:** 2025-10-17

**Authors:** Asher R. Utz, Linlin Ma, Dalton Hilovsky, Brenna M. Zimmer, Emily Allego, Jade Fluharty, Pooja Narasimhan, Jeffrey R. Enders, George Grady, Monica Milici, Pengda Liu, Xiaojing Liu, Joseph J. Barycki, Melanie A. Simpson

**Affiliations:** a Department of Molecular and Structural Biochemistry, North Carolina State University, USA; b Molecular Education, Technology and Research Innovation Center, North Carolina State University, USA; c Department of Biochemistry and Biophysics, University of North Carolina - Chapel Hill, USA

**Keywords:** Prostate cancer, Cell growth, Cell motility, Hyaluronan, Glycosaminoglycan, N-linked glycosylation, O-linked glycosylation, Phosphorylation, Dehydrogenase

## Abstract

UDP-glucose 6-dehydrogenase (UGDH) is an essential enzyme that catalyzes the oxidation of UDP-glucose to UDP-glucuronate. UGDH is elevated in multiple cancers, including prostate cancer, and is functionally implicated in castration resistant recurrence. UGDH is composed of three dimeric units that associate stably as a hexamer in cellular conditions. The dynamic reorganization of noncovalent interactions at the dimer contact interfaces is essential for UGDH activity. In this study, we examined the functional relevance of a putative AGC kinase motif located at the dimer-dimer interface. We demonstrated that UGDH is phosphorylated in LNCaP cells, specifically at serine 316, by RSK2, p70S6K, and SGK1. To determine the functional implications of UGDH S316 phosphorylation, we generated and characterized phosphomimetic (S316D) and phosphodeficient (S316A) point mutations. Intrinsic properties of the purified recombinant proteins were only modestly affected by the substitutions. The stable overexpression of UGDH S316D in LNCaP cells significantly increased the rate of N- and O-glycan synthesis, as well as the production of hyaluronan and sulfated glycosaminoglycans, while reducing DHT glucuronidation, resulting in significant increases in growth of tumor spheroids, cell proliferation and motility, and resistance to enzalutamide. In contrast, UGDH S316A expression reduced the production of glycans and glycosaminoglycans, restored DHT glucuronidation, and impaired growth and motility. Overall, our results support UGDH phosphorylation as a point of control for intracellular and cell surface glycan production, thereby regulating cell proliferation, anchorage dependence, motility, and tumor cell therapeutic resistance.

## Introduction

Prostate cancer is a leading cause of male cancer death [1]. Mechanisms underlying the aggressive progression and development of therapeutic resistance during treatment of prostate cancer by androgen deprivation are areas of active research. Among those most heavily studied are the inappropriate expression of androgen biosynthesis enzymes in the tumor cells, overexpression or mutation of the androgen receptor (AR), and the emergence of AR splice variants that engage alternative patterns of gene expression independently of androgens [2–4]. Thus, a more detailed understanding of cellular metabolite sensing and consequent reprogramming of cell phenotype is needed to improve response.

Castration resistant prostate cancer (CRPC) cell lines and patient-derived xenografts selected for castration resistance express higher levels of UDP-glucose dehydrogenase (UGDH) relative to those dependent on exogenous or circulating androgens [5,6]. The overexpression of UGDH causes altered use of nucleotide sugar precursors to support biosynthetic processes such as the synthesis of glycosaminoglycans (GAGs) and proteoglycans at the expense of glucuronidation, the process by which excess androgens are targeted for cellular elimination [6–9]. This switch results in a decrease in androgen-glucuronide output and promotes both tumor growth and resistance to the anti-androgen enzalutamide (enz). In contrast, the knockdown of UGDH reduces the expression of Notch1, a plasma membrane receptor dependent on UGDH for precursors of the glucose-xylose-xylose trisaccharide modification needed for cell surface expression [10], and partially restores the sensitivity to enz, presumably by re-establishing and sustaining androgen dependence via altered glucuronidation [5]. However, this uncoupling of processes was an unexpected finding that suggested there could be a mechanism by which UGDH could sense different needs of the cell and establish a priority for directing the fates of nucleotide sugars.

**Fig. 1.**
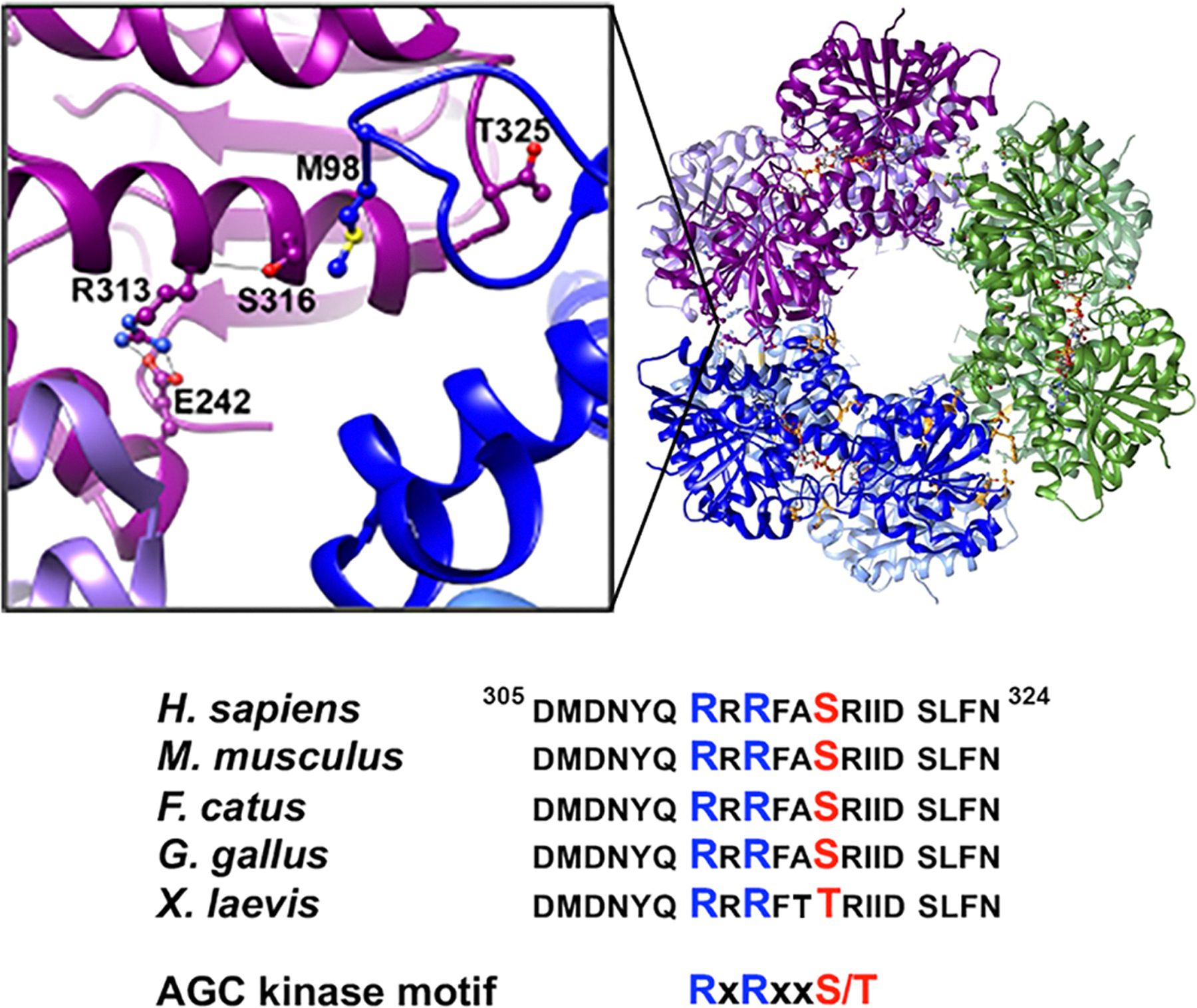
UGDH bears a conserved consensus sequence for phosphorylation by members of the AGC kinase family. Ribbon representation of the UGDH homohexamer with bound substrate and cofactor depicted in ball and stick representation is colored by subunit to indicate quaternary assembly as a trimer of dimers (pdb 2Q3E). Respective dimeric units are purple/light purple, blue/light blue, and green/light green. A zoomed view of the interface between dimers is shown with side chains of significant residues involved in structural integrity of the interface shown as ball and stick, including S316. The specific sequence that comprises the consensus for AGC kinases in UGDH from *Multiple species* is conserved as shown. Although not present in the alignment, the adjacent residue, T325, is also fully conserved in these species.

UGDH catalyzes the oxidation of UDP-glucose (UDP-Glc) to UDP-glucuronate (UDP-GlcA) [11]. The potential for UGDH to act as a metabolic sensor is supported by its well characterized structural properties and the connection to aspects of its allosteric control *in vitro* as well as its ability to direct UDP-sugar fates in the cell [12–20]. Notably, UGDH is a homohexameric enzyme that assembles as a trimer of dimers (Fig. 1). The dynamic reorganization of hydrogen bond networks at the interface between dimeric units is required to allow for initial binding of substrate and the cofactor NAD^+^, as well as the mid-reaction exchange of reduced for oxidized cofactor, and ultimately the release of the products. UDP-xylose (UDP-Xyl), a potent competitive inhibitor of UGDH activity, induces a conformation in the UDP-Xyl–-bound crystal structure that features one open interface between dimeric units [16]. Maximal enzymatic function is lost when the dimer-dimer network is disrupted either by mutagenesis or chemical cross-linking [15,21]. This movement may provide an opportunity for differential post-translational modifications that could impact UGDH activity in a reversible manner, including phosphorylation events that could regulate UGDH in response to the prevailing extracellular conditions. A role for phosphorylation of UGDH in altering cell processes has been previously shown. Tyrosine Y473 in the C-terminal domain of UGDH can be phosphorylated; however, this was related to its relocation from the cytosol to the nucleus with no effect on enzymatic activity in the cell [22].

Since UDP-GlcA is required for divergent functional outcomes that occur in extranuclear compartments of the cell, namely Golgi-localized proteoglycan synthesis and ER luminal androgen glucuronidation, we hypothesized that UGDH would be a point of regulation for metabolite distribution. In the current study, we located a phosphorylation consensus sequence at the interface between dimeric subunits of the UGDH homohexamer. Our first goal was to determine whether this was a functional phosphorylation site, identify one or more kinases that were responsible for phosphorylation and confirm the residue that was phosphorylated. We subsequently generated phosphomimetic and phosphodeficient point mutants in the UGDH sequence and characterized the impacts on the physical properties of the UGDH enzyme, as well as the consequences in cellular function and downstream fates. Our results support a model in which the phosphorylation of UGDH optimizes use of UDP-GlcA in biosynthetic pathways directly and indirectly increasing glycans and glycosaminoglycans. The consequence is a significant increase in intrinsic proliferation of the cells and promotion of anchorage independent growth and motility.

## Results

### The UGDH dimer-dimer interface contains a functional consensus sequence for phosphorylation by members of the AGC kinase family

UGDH is a homohexamer organized as a trimer of dimers. The dimer-dimer interface of UGDH has been shown to require dynamic motion to accommodate cofactor switching during catalysis. Mutagenesis of residues at the dimeric interface that disrupt these conserved non-covalent interactions results in reduced steady state activity [15]. Moreover, the recombinant UGDH T325D mutant purifies as a dimer that cannot be induced to adopt the hexameric state by substrate and cofactor addition unlike the UGDH wild-type (WT) enzyme. This dimeric mutant has significantly reduced activity *in vitro* and in cells [15]. Sequence alignment of UGDH from several species revealed the presence of a consensus motif for phosphorylation by members of the AGC kinase family of serine/threonine kinases, RxRxxS/T, where x can be any amino acid [23, 24] (Fig. 1). The motif spans residues 311–316 of the human sequence, which is a region located within the dimer-dimer interface of the homohexameric enzyme in proximity to T325, a residue critical for hexameric assembly (Fig. 1).

**Fig. 2.**
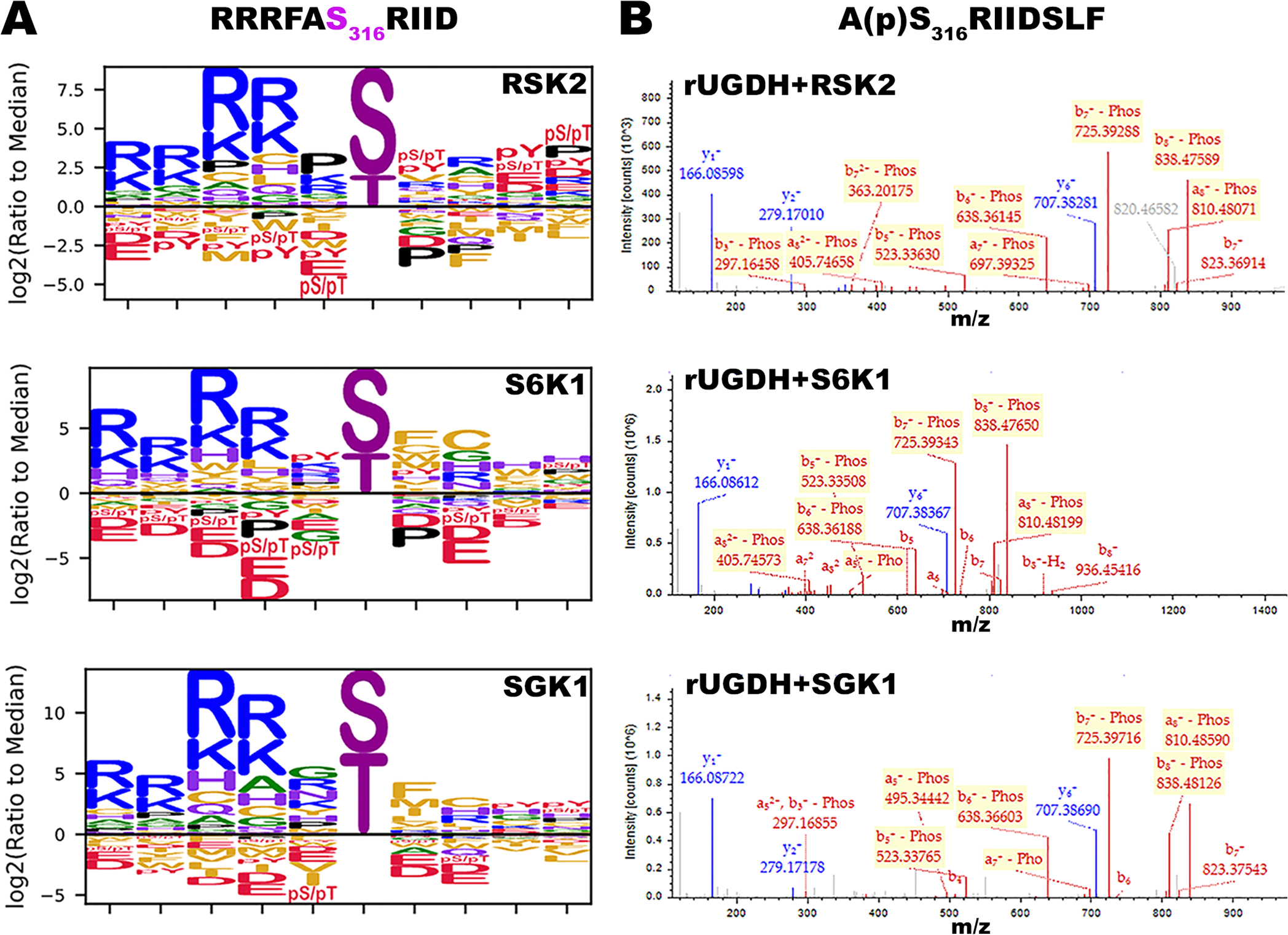
Identification of AGC kinases that phosphorylate UGDH S316. A) Consensus sequences for targets of RSK2 (RPS6KA3), p70S6K (aka S6K1 or RPS6KB1), and SGK1 were generated using PhosphoSitePlus [23]. The specific sequence corresponding to this consensus in UGDH is given at the top of column A. B) Following *in vitro* kinase assays with purified recombinant human UGDH and the indicated kinase, chymotrypsin-digested peptides were analyzed by LC-MS/MS to detect presence or absence of the S316-containing phosphopeptide fragment. The sequence of the detected fragment is given at the top of column B.

To determine if serine 316 (S316) could be phosphorylated and by which members of the AGC kinase family, we performed an *in vitro* kinase screen with AGC kinases that were predicted to phosphorylate target proteins bearing consensus sequences matching that of the UGDH S316 peptide (Fig. 2A). Human recombinant UGDH was expressed and purified from *Escherichia coli* (with no phosphorylation) and incubated with each activated recombinant human kinase. The presence of the phosphorylated UGDH peptide containing pS316 was analyzed by liquid chromatography and tandem mass spectrometry (LC-MS/MS). Of the AGC kinases screened, RPS6KA3 (RSK2), RPS6KB1 (p70S6K or S6K1), and SGK1 (serum and glucocorticoid-regulated kinase 1) produced a peptide fragment whose sequence corresponded to the digestion product predicted to contain UGDH pS316 (Fig. 2B). We additionally screened RSK3, MELK, STK33, ERK2, and PIM2, none of which yielded a detectable pS316 peptide (data not shown).

**Fig. 3.**
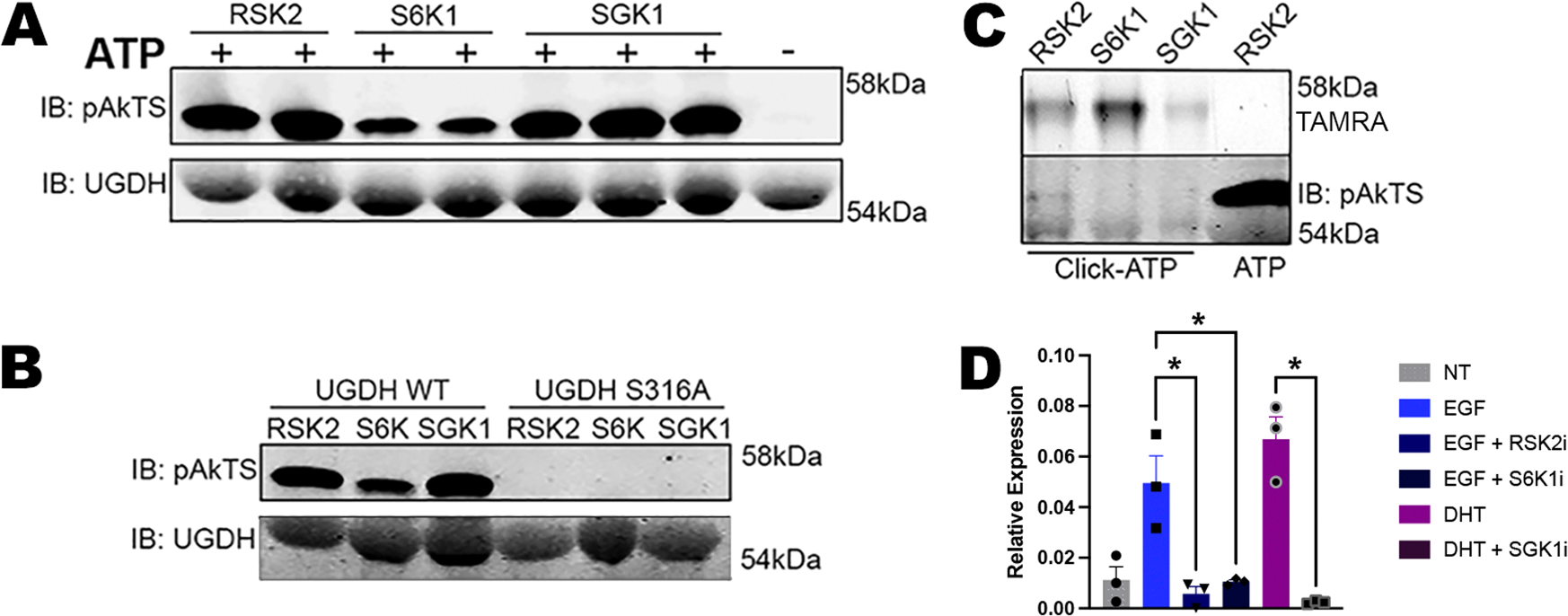
Phosphorylation of UGDH S316 in cells is stimulated by EGF and DHT downstream of RSK2, S6K1, and SGK1 in LNCaP cells. A) Recombinant human UGDH was incubated with the indicated kinases in the presence or absence of ATP and analyzed by western blot probed for UGDH pS316 using anti-phospho-AkT substrate antibody or total anti-UGDH. B) UGDH WT or S316A phosphodeficient mutant was incubated with the indicated kinases and analyzed by western blot. C) UGDH WT was incubated with indicated kinases with “clickable” or standard ATP. The clickable phosphate was detected by conjugation to TAMRA followed by SDS-PAGE. D) LNCaP cells were stimulated with either EGF or DHT, followed by treatment with vehicle control or the indicated kinase inhibitor. Whole cell lysates were analyzed by western blot probed for anti-p-AkT substrate. Results were quantified by densitometry normalized to tubulin and plotted as mean ± SEM for three biological replicates. Individual data points are shown; *p < 0.01.

To validate the phosphorylation of UGDH by RSK2, S6K1, and SGK1 in a cellular context, we used a western analysis that capitalized on the general reactivity of an antibody generated against the phosphorylated AGC kinase motif (RxRxxpS/T) for targets of Akt, which is also a member of this kinase family. To validate the assay, we first compared phosphorylation of S316 in the *in vitro* kinase assay using purified recombinant UGDH. Each of the three kinases produced a band of the expected molecular mass for p-UGDH analyzed by western blot (Fig. 3A and SFig 1). The band corresponding to UGDH pS316 was absent in the UGDH S316A mutant (Fig. 3B and SFig 1), and also the UGDH S316D mutant (not shown), verifying the use of serine 316 as a specific phosphorylation site. To confirm this with an independent method, we performed *in vitro* kinase assays with a clickable synthetic ATP analog [25], γ-[(6-Azidohexyl)-imido]-ATP, which we conjugated to the tetramethylrhodamine fluoroprobe TAMRA. The fluorescent TAMRA-labeled band electrophoresed at the identical molecular mass of p-UGDH detected as the pAkt substrate band in western blot analysis, confirming this band corresponds to phosphorylated UGDH (Fig. 3C and SFig 1). To observe phosphorylation of UGDH at this motif in cells, we immunoprecipitated UGDH from LNCaP cell lysates and immunoblotted with anti-pAkt substrate, which revealed a single band corresponding to the molecular mass of UGDH (SFig. 1). We further validated using serum starved LNCaP cells treated with EGF to stimulate activity of RSK2 [26] and S6K1 [27], or DHT to activate SGK1 [28], prior to lysate harvest. Strong stimulation of UGDH phosphorylation was detected in whole cell lysates as expected (Fig. 3D and SFig 1). EGF-stimulated band intensity was significantly reduced by specific inhibitors of RSK2 (BIX02565 [29]) and S6K1 (PF4708671 [30]), and the DHT-stimulated band was virtually eliminated by specific inhibition of SGK1 with GSK650394 [28] (Fig. 3D and SFig 1). Reduction of this signal by treatment with pharmacological agents blocking each of the three kinases in a relevant cellular context strongly supports UGDH S316 as a novel target of phosphorylation through these pathways.

Due to the location of UGDH S316 at the dimeric interface, we tested whether UGDH oligomeric status influenced the ability of each kinase to phosphorylate UGDH S316. In vitro kinase assays were performed to compare phosphorylation of UGDH S316 in the WT enzyme versus the UGDH T325D obligate dimer mutant. All three kinases produced a phosphorylation band in the dimeric UGDH T325D as expected. The presence of the endogenous inhibitor UDP-Xyl, which stabilizes an alternative conformation of the UGDH enzyme, did not impact phosphorylation (data not shown).

### Phosphomimetic UGDH S316D mutation does not significantly alter the enzyme characteristics in vitro

UGDH S316 is located within the same element of secondary structure as T325. The mutation of T325 to aspartate was previously shown to result in an obligate dimeric quaternary assembly of UGDH, with 80 % loss of steady state catalytic activity [15]. Therefore, we considered the possibility that phosphorylation of UGDH at S316 could impact its function by interfering with critical allosteric dynamics and/or hexamer stability. We tested this by introducing point mutations to S316, substituting serine with aspartate (S316D) to produce a phosphomimetic version of UGDH, or with alanine (S316A) to simulate a phosphodeficient state. We then compared the resultant UGDH quaternary structure and catalytic activity to the WT or T325D obligate dimeric species.

We first generated the UGDH S316 point mutants in a bacterial construct that expressed the proteins with a six-histidine fusion to facilitate purification by nickel affinity chromatography as we have done previously [14,15,21,31,32]. Protein yields for UGDH S316A were comparable to those of WT UGDH. However, UGDH S316D yields were somewhat lower as a result of protein precipitation following purification. To compare the intrinsic stability of the S316 point mutants, we used a modified version of the Thermofluor Stability Assay [33], which we have used previously as a preliminary indicator of UGDH quaternary assembly [14,15,21,34]. The UGDH WT and UGDH S316A enzymes had significant increases in melting temperature (T_m_) upon addition of either substrate or cofactor and were stabilized by almost 20°C in the holoenzyme complex as expected. The UGDH S316D mutant had a modestly lower T_m_ in all conditions relative to the WT UGDH, suggesting slightly reduced stability of this mutant (Table 1). Overall, both UGDH S316D and UGDH S316A mutants were significantly more stable than the previously reported obligate dimer UGDH T325D.

A comparison of steady state kinetics among UGDH S316A, S316D, and the WT and T325D controls revealed similar steady state catalytic activity of UGDH S316A relative to WT UGDH (Table 1). When the concentration of the substrate UDP-Glc was varied with saturating NAD^+^, UGDH S316A and UGDH S316D showed a K_m_ for UDP-Glc of 36 μM and 19 μM, respectively, and a V_max_ of 550 nmol/min/mg and 310 nmol/min/mg, respectively (Table 1). When the cofactor NAD^+^ was the variable, UGDH S316A and UGDH S316D showed a K_m_ of 380 μM and 330 μM, respectively, and a V_max_ of 490 nmol/min/mg and 230 nmol/min/mg, respectively (Table 1). For WT UGDH, the K_m_ for UDP-Glc was 12 μM with a V_max_ of 640 nmol/min/mg, and the K_m_ for NAD^+^ was 530 μM with a V_max_ of 710 nmol/min/mg (Table 1). Therefore, the V_max_ values for UGDH S316D were ≈two-fold lower than those of WT UGDH. In contrast, we previously reported the V_max_ values for the UGDH T325D obligate dimer as ≈65–75 nmol/min/mg [15].

We further examined quaternary structure of the point mutants using gel filtration chromatography. The elution profiles of UGDH S316D and S316A had two peaks that superimposed well with those of the hexameric and dimeric forms of WT UGDH (SFig 2A). The dimer and hexamer peaks were confirmed by similar elution time of the UGDH T325D dimeric and UGDH Δ132 hexameric mutants (SFig 2B). As seen for UGDH WT, both mutants were fully stabilized to a hexamer by addition of substrate and cofactor to form the holocomplex (SFig 2C). As a final indicator of the stability of UGDH S316 mutants, we performed limited trypsin digestion (SFig 2D). Modestly increased susceptibility to proteolytic degradation was observed for UGDH S316D in the apo and holo forms relative to UGDH WT. Collectively, the overall quaternary assembly and intrinsic stability were not grossly altered in either point mutant.

Finally, we tested whether the S316 point mutants impacted inhibition of UGDH by UDP-xylose (UDP-Xyl), which is an endogenous competitive inhibitor of UGDH with nM affinity that causes a conformational change in the crystal structure exposing one of the dimer interfaces [20]. We measured the concentration dependence of UDP-Xyl inhibition in the context of the catalytic reaction at sub-saturating UDP-Glc and NAD^+^ and used the resultant curves to calculate the IC_50_ for UGDH S316A and S316D (Table 1). The IC_50_ values for each point mutant were comparable to the IC_50_ of WT UGDH. The presence of UGDH S316A and S316D point mutations also did not impact UGDH thermal stability in the presence of UDP-Xyl (data not shown). Binding of UDP-Xyl to UGDH independently of catalysis was measured by ITC. The K_d_ for UGDH S316D was ≈2-fold higher than the K_d_ values for UGDH WT and S316A (150 nM versus 91 and 71 nM, respectively). Although the IC_50_ for UDP-Xyl was not different, the Kd reflects intrinsic affinity whereas the IC_50_ is sensitive to culture conditions, which in this case would not likely challenge the competitive binding of UDP-Glc versus UDP-Xyl. Altogether, we predicted the impact of phosphorylation at UGDH S316 could be context-dependent in the cell and regulate metabolic outcome in a manner consistent with the directives of external stimuli, since its intrinsic properties were minimally affected by phosphomimicry.

### Stable expression of the UGDH S316D phosphomimetic mutant in LNCaP prostate tumor cells increases UGDH activity and alters UDP-sugar levels

**Fig. 4.**
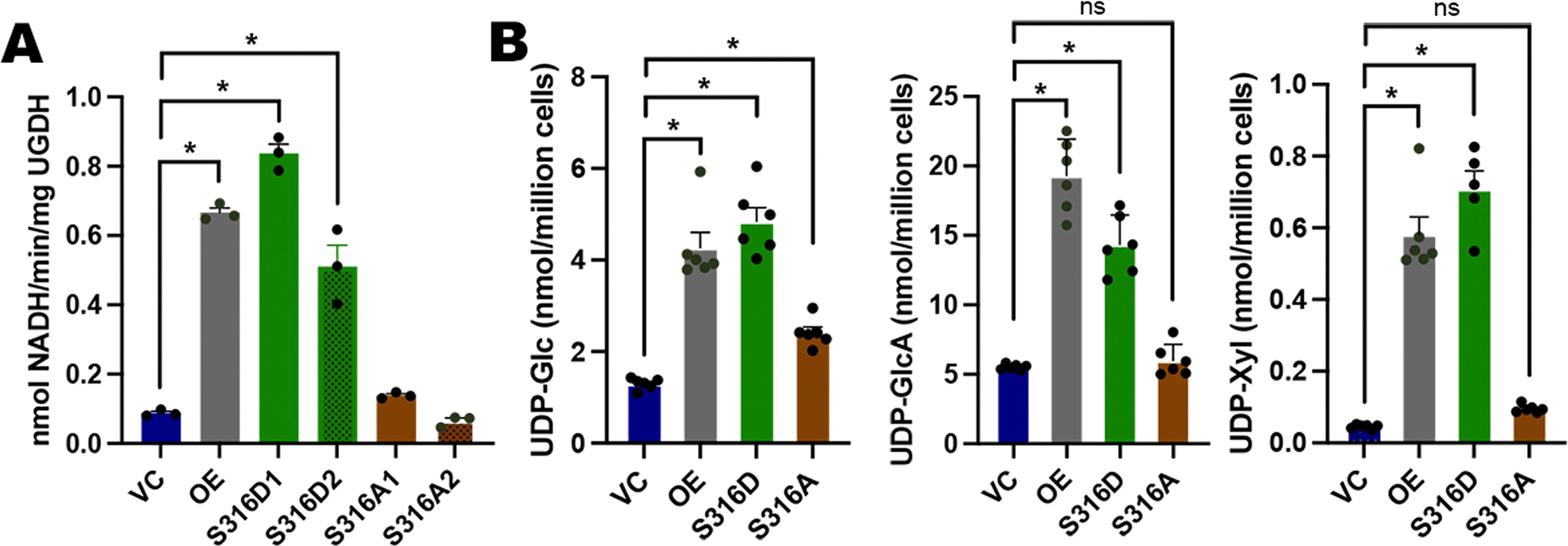
LNCaP cells selected for stable overexpression of the UGDH S316D phosphomimetic mutant have significantly increased UGDH activity reflected in increased UDP-sugar flux. Whole cell lysates from LNCaP cells expressing control (VC), UGDH WT (OE), UGDH S316D or S316A mutants were assayed for (A) UGDH activity by monitoring increased fluorescence of NADH, or (B) levels of UDP-Glc, UDP-GlcA, and UDP-Xyl by LC-MS/MS. All values are plotted as mean ± SEM for three replicates with individual data points shown. * *p* < 0.01.

To test the selectivity of UGDH phosphorylation with respect to the downstream fates of the UDP-GlcA product, we first evaluated whether simulating UGDH S316 phosphorylation would alter UGDH enzymatic activity in living cells. LNCaP prostate tumor cell lines were stably selected for overexpression of an empty vector control (designated VC), UGDH WT (designated OE), UGDH S316D (two clonal lines S316D1 and S316D2), or UGDH S316A (two clonal lines S316A1 and S316A2). Whole cell lysates were prepared from each of the lines and UGDH activity was assayed following incubation of each lysate with UDP-Glc and NAD^+^ as we have done previously [34]. Cells expressing either UGDH WT or the phosphomimetic UGDH S316D had ≈6–8 fold increased UGDH activity relative to VC cells (Fig. 4A). In contrast, expression of UGDH S316A in LNCaP cells did not confer UGDH activity above the endogenous levels seen in the VC cells.

We then compared the levels of UDP-sugars that constitute the substrate, product, and feedback inhibitor of UGDH. Overexpression of UGDH WT (OE) or S316D significantly increased UDP-Glc levels (≈4–5-fold) and flux through UGDH and UXS1 as indicated by concomitant increases in the respective products, UDP-GlcA (≈3–4-fold) and UDP-Xyl (≈6–7-fold, Fig. 4B). Cells expressing UGDH S316A had modestly (≈2-fold) increased UDP-Glc. However, in contrast to the UGDH WT and S316D cell lines, UDP-GlcA and UDP-Xyl levels were unchanged by expression of the phosphodeficient UGDH S316A mutant relative to those in cell lysates expressing only the vector control, consistent with the unchanged activity of UGDH in cell lysates of VC and UGDH S316A expressing cells.

### UGDH S316D expression increases cellular capacity for glycan synthesis and promotes the use of UDP-GlcA in glycan production

**Fig. 5.**
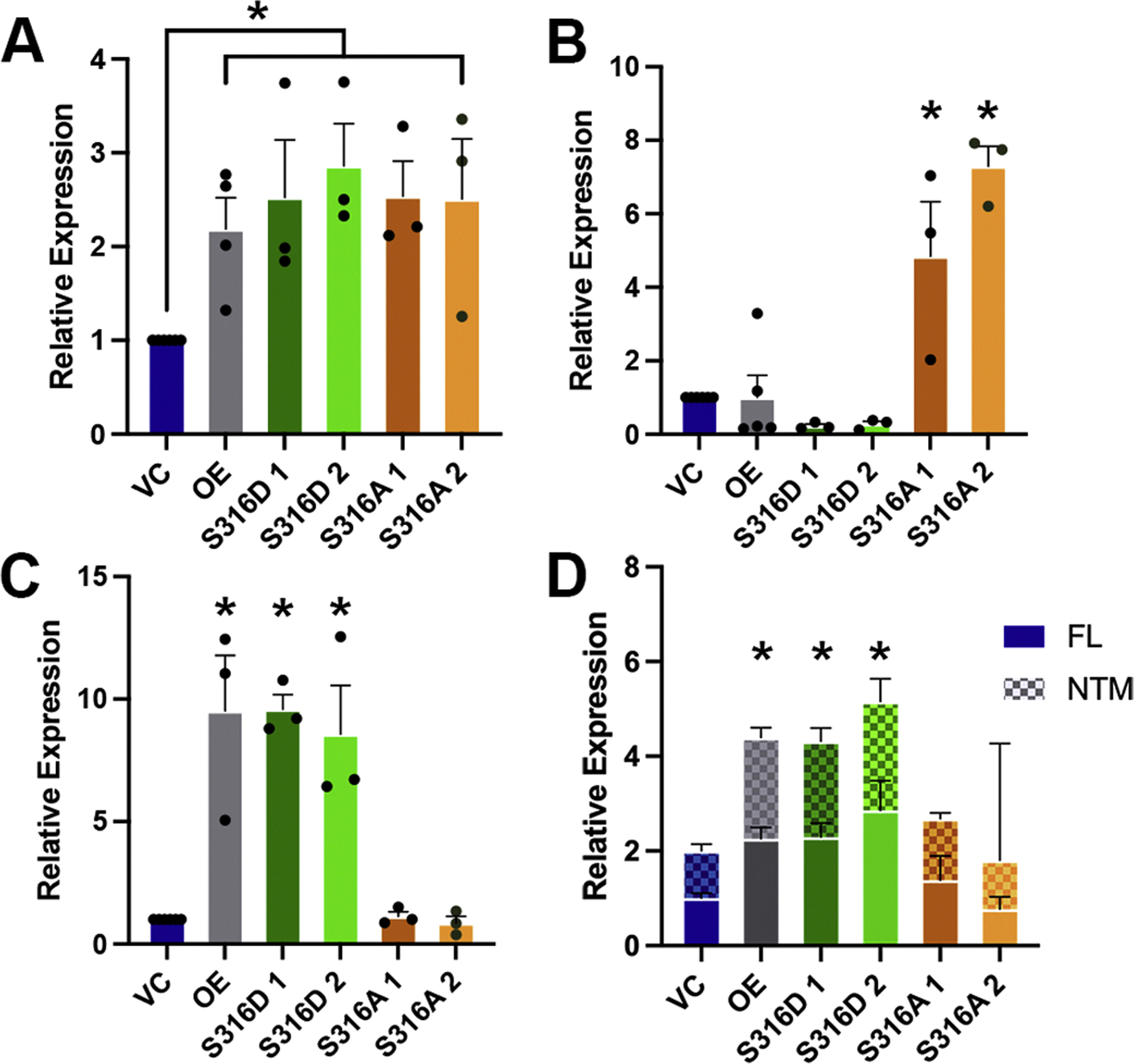
Phosphomimetic UGDH expression increases levels of UXS1 and Notch1. Whole cell lysates were analyzed by western blot from LNCaP cells selected for stable expression of vector control (VC), or constructs overexpressing UGDH WT (OE), UGDH S316D, or UGDH S316A (two clonally selected lines each for the mutants). Westerns were probed for expression of (A) UGDH, (B) UGT2B17, (C) UXS1, or (D) Notch 1 full length (FL) and N-terminal and membrane domain (NTM). Mean ± SEM is plotted with individual data points shown, and significant differences between UGDH OE and VC or UGDH S316 mutants relative to VC are indicated by an asterisk; * *p* < 0.01.

To compare the impacts of UGDH S316 phosphorylation mutants further, we next examined expression of specific genes involved in the downstream pathways utilizing UDP-GlcA. Immunoblot analysis confirmed UGDH overexpression in the UGDH WT (OE), UGDH S316D, and UGDH S316A cell lines relative to the VC cell line as expected (Fig. 5A and SFig 3). We then checked the level of UGT2B17, one of the glucuronosyltransferases responsible for androgen glucuronidation in the prostate, expression of which we previously showed was insensitive to DHT suppression when UGDH WT was overexpressed [5]. As expected, cells overexpressing UGDH WT (OE) had comparable levels of UGT2B17 relative to the VC line. However, while UGDH S316A cell lines had elevated UGT2B17 expression consistent with heightened glucuronidation potential, UGDH S316D cell lines had comparable or slightly diminished UGT2B17 protein levels (Fig. 5B and SFig 3). Interestingly, the expression of UDP-xylose synthase 1 (UXS1) was ≈6–8-fold elevated in UGDH WT or S316D-expressing cells, but was comparable in UGDH S316A lines relative to the VC line (Fig. 5C and SFig 3). This result is consistent with the levels of UDP-Xyl measured in the lysates (Fig. 4B), and suggested the potential for significantly increased flux of UDP-GlcA into the production of glycans, sulfated glycosaminoglycans (sGAG) and proteoglycans. Therefore, we also compared the expression of Notch1 on the cell surface as a measure of ER and Golgi UDP-Xyl availability, which is needed for Notch1 trisaccharide modification prior to presentation at the cell membrane [10]. Levels of both full length (FL) and the cleaved N-terminal transmembrane portion (NTM) of Notch1 were lower in the UGDH S316A lines relative to the LNCaP VC cells (Fig. 5D). In contrast, the expression of Notch1 FL and Notch1 NTM were significantly elevated in UGDH WT (OE) and S316D cell lines (Fig. 5D).

**Fig. 6.**
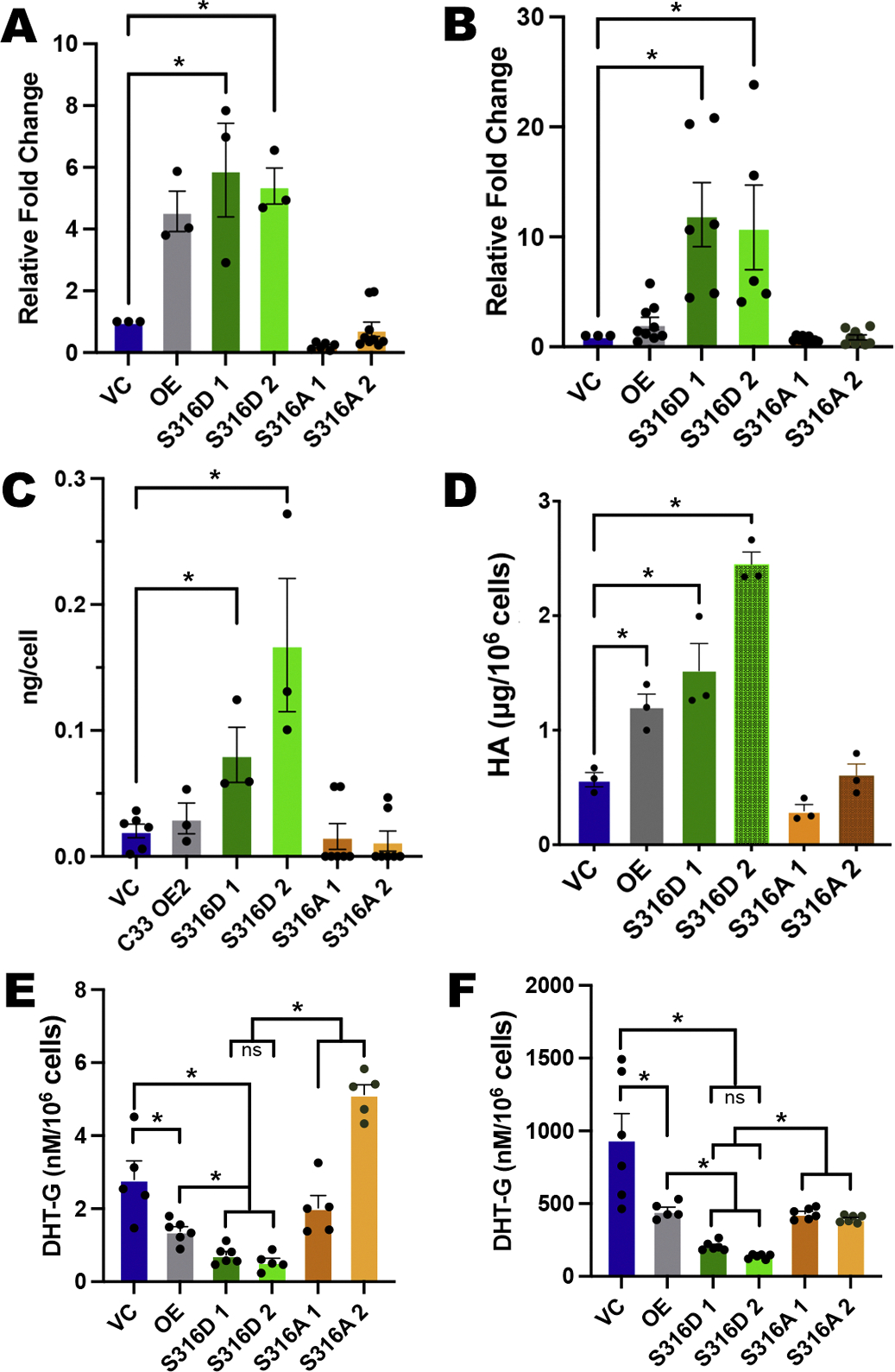
Overexpression of phosphomimetic UGDH increases the rate of protein N- and O-glycosylation, as well as the production of HA and sulfated GAGs, but decreases the secretion of DHT-glucuronide. Glycans were quantified by click chemistry following incubation of each cell line as indicated with azido-labeled precursor sugars for A) O-glycans and B) N-glycans. C) The sulfated GAGs were enzymatically dissociated from each line and colorimetrically detected by SDS-PAGE fractionation and digital image analysis. D) HA was quantified by competitive binding assay of conditioned media from the indicated cells normalized to cell count. DHT-glucuronide (DHT-G) was quantified by LC-MS in extracts of conditioned media from the indicated cells cultured continuously in standard media E) or in androgen depleted media followed by addition of 10 nM DHT (F). For each assay, mean ± SEM is plotted for at least three replicates with individual points shown. Significance is indicated; * *p* < 0.01.

We then sought to evaluate changes to biosynthetic pathways on a global scale, beginning with glycan synthesis. To do this, we employed bioorthogonal click chemistry to detect nascent synthesis of O-linked and N-linked glycans in living cells. Cells were incubated with modified azido sugars serving as analogs for GalNAc, N-azidoacetylgalactosamine (GalNAz), and GlcNAc, N-azidoacetylglucosamine (GlcNAz), which have been shown to label O- and N-glycans, respectively [35,36]. Cells overexpressing UGDH WT had ≈4-fold higher O-glycan and comparable N-glycan labeling relative to the VC line (Fig. 6A, Fig. 6B, and SFig 4). The UGDH S316D cell lines had ≈6-fold higher O-glycan and ≈10-fold more N-glycan than the VC cells, which was significantly more than the UGDH WT (OE) levels. In contrast, glycan labeling in the UGDH S316A lines was comparable to the VC cells. Incorporation of GlcNAz into N-glycans in the UDGH S316D lines was confirmed by the sensitivity of the signal to removal of N-glycans by PNGase F digestion (SFig 4B). Treatment of UGDH WT (OE) cells with the UDP-GlcA scavenger, 4-methylumbelliferone (4-MU [37]), in conjunction with metabolic O-glycan labeling reversed the enhancement of O-glycan levels (SFig 5). However, this reversal was not observed in UGDH S316D cell lines, suggesting a compensatory mechanism that maintains UDP-GlcA partitioning to biosynthesis even when intracellular availability is reduced.

Cells overexpressing UGDH S316D also generated significantly more sGAG, which we measured via dye-binding assay after enzymatic release from the cell surface proteoglycan pool [38] (Fig. 6C). The production of sGAG by UGDH S316D cell lines was ≈3–5-fold higher than the VC or OE lines. UGDH S316D cell lines also produced and secreted ≈3–5-fold more hyaluronan (HA), which was quantified in the cell conditioned media [7,15,39,40], relative to the VC line and 30–100 % more than the UGDH WT (OE) line (Fig. 6D). Production of sGAG and HA by UGDH S316A was unchanged relative to the VC line. These findings are consistent with UGDH S316 phosphorylation serving as a regulatory node for multiple important glycan biosynthetic processes.

We then investigated the third fate of UDP-GlcA by measuring the androgen glucuronide output of the cells. When cultured in standard media (which are androgen replete because of the presence of serum), the UGDH WT (OE) line secreted ≈50 % of the level of DHT-glucuronide (DHT-G) that was secreted by the VC line (Fig. 6E). The UGDH S316D lines both produced <20 % of the amount of DHT-G found in media of VC cells (Fig. 6E), consistent with significantly reduced expression of UGT2B17 (Fig. 5B) in the UGDH S316D lines. In contrast, UGDH S316A lines secreted as much or more DHT-G as the VC line (Fig. 6E), consistent with their increased UGT2B17 level (Fig. 5B). To test the extent to which each line retained its sensitivity to DHT, we additionally measured the secreted DHT-G levels of cells that were first androgen depleted, followed by reexposure to DHT. Identical trends were observed, with UGDH WT (OE) cells secreting 50 % less DHT-G, and UGDH S316D lines secreting <20 %, relative to the VC line. UGDH S316A lines, upon DHT stimulation, produced similar levels of DHT-G to the UGDH WT (OE) line. These results are consistent with an important role for UGDH phosphorylation in maintaining cellular glucuronidation, in a manner that is inverse to its impact on biosynthesis, further implicating the phosphorylation event as a control point.

### UGDH S316D expression dramatically increases tumor cell proliferation in a kinase dependent manner that supports tumor spheroid growth and cell motility

**Fig. 7.**
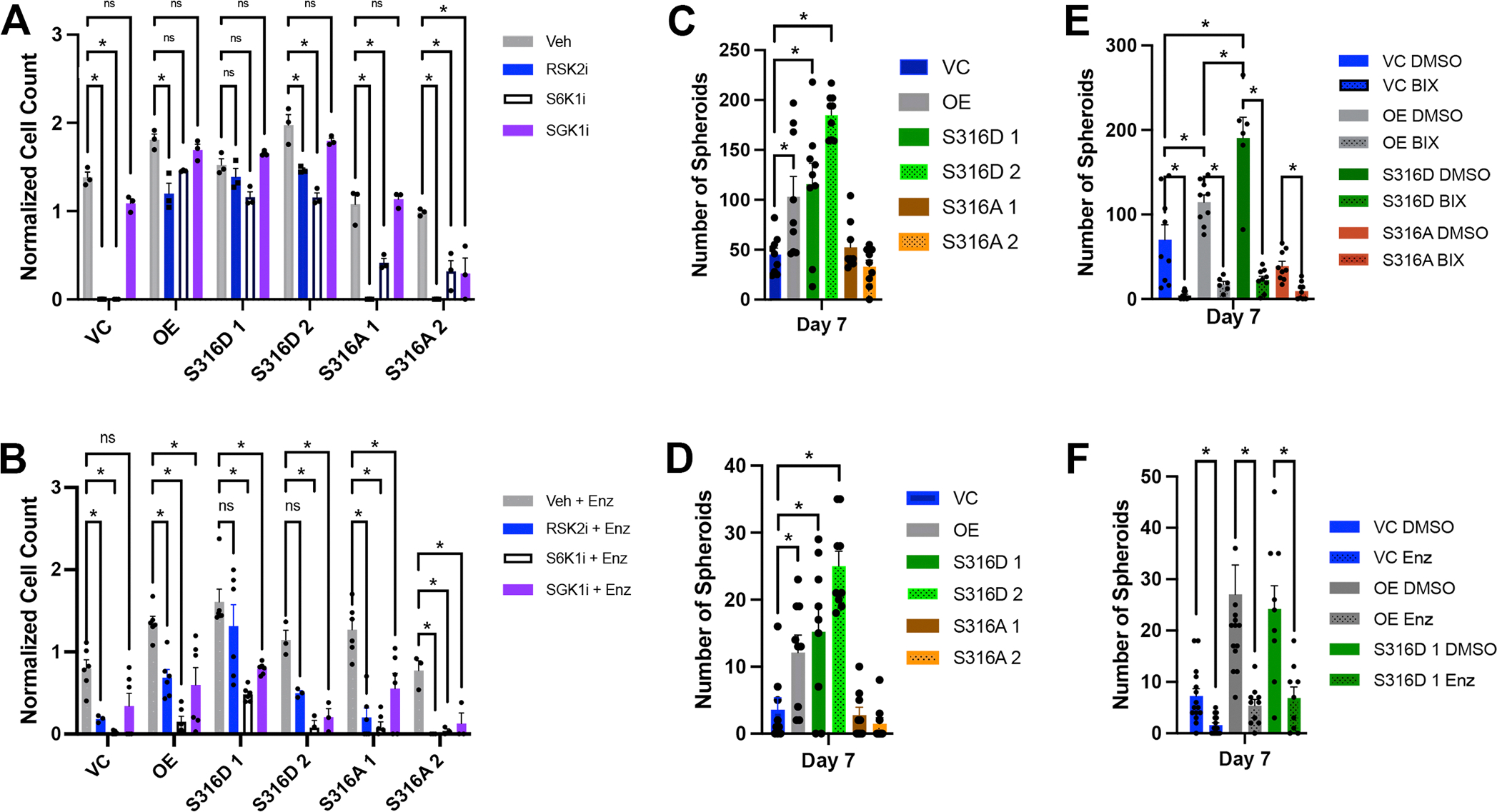
UGDH S316D expression promotes uncontrolled cell proliferation, tumor spheroid growth, and enzalutamide resistance. Cells were assayed for 3-day proliferation rates in the absence or presence of the indicated inhibitors by resazurin conversion to resorufin either without (A) or with (B) the additional treatment with enz. Medium (C) and large (D) spheroid counts were compared among lines before testing effects of the RSK2-specific inhibitor BIX02565 (E) and the anti-androgen agent enzalutamide (enz, F). Mean ± SEM is plotted for at least three replicates with individual points shown. Asterisks denote significance in the pairwise combinations indicated * *p* < 0.01; ns, not significant. Also significant but not indicated on the figure are comparisons between treatments in the absence (A) versus presence (B) of enz for VC, S316A1 and S316A2. UGDH OE, S316D1, and S316D2 proliferation in the presence of enz (B) was not significant relative to the respective vehicle treatments (A).

We previously found that UGDH WT overexpression promoted proliferation and blunted response to the anti-androgen enzalutamide (enz) in LNCaP cells [5] so we compared these processes in the cells expressing phosphorylation mutants. As expected, the overexpression of UGDH WT (OE) increased cell proliferation by ≈30 % (Fig. 7A, compare vehicle-treated conditions, gray bars). This effect was also observed in UGDH S316D lines, but the growth of UGDH S316A lines was unchanged or slightly reduced relative to the VC cells. We then tested the relevance of the AGC kinases to altered cell proliferation. Cells were treated with specific inhibitors of RSK2, S6K1, and SGK1 or a vehicle control to determine the degree to which each inhibitor would affect proliferation, expecting that the UGDH S316D and UGDH S316A lines would have different responses than the UGDH WT (OE) line. The RSK2 inhibitor BIX02565 reduced cell number in all lines, and induced significant cell death in VC and UGDH S316A cells (Fig. 7A). Blocking S6K1 activity with the specific inhibitor PF4708671 also reduced proliferation in all lines, with the most dramatic impacts occurring in VC and UGDH S316A lines, likely reflecting effects on the endogenous UGDH protein (Fig. 7A). SGK1 inhibition with GSK650394 did not abrogate cell proliferation in replete medium (not shown). However, significant impacts were observed when cells were serum and steroid depleted for 48 h prior to the addition of serum and the requisite 10 nM DHT for SGK1 activation just prior to inhibitor treatment (Fig. 7A). Overall, the level of response measured in UGDH S316D lines was similar to the UGDH WT (OE) cells for each of the inhibitors.

We additionally wanted to know whether the inhibition of AGC kinases could sensitize the antiproliferative response to enz treatment. Individual inhibition of each kinase in tandem with enz treatment significantly reduced cell number compared to enz treatment alone across all cell lines (Fig. 7B). Growth of VC and UGDH S316A-expressing cells was suppressed by ≈40 % as previously observed for LNCaP cells [5]. However, although growth was reduced, the UGDH WT and S316D cells continued to proliferate in the presence of enz alone (Fig. 7B, compared to Fig. 7A, vehicle). Targeting all three kinases concurrently significantly reduced cell number in all cell lines and was more effective than enz treatment alone (SFig 6). The combined targeting of RSK2 with enz eliminated growth of VC and UGDH S316A lines (Fig. 7B). This treatment also reduced growth of UGDH WT and S316D by ≈20 %. The most significant impacts were observed by inhibition of S6K1 or SGK1 concurrently with enz. Only the growth of UGDH S316D lines remained significant in the presence of enz and S6K1 inhibitor, but was reduced to <30 % of initial cell count. SGK1 inhibitor combined with enz also suppressed growth of all lines to <40 %. These results support a potential role for UGDH phosphorylation in promoting resistance to enz and other therapeutic agents by elevating glycans downstream of AGC kinase activation.

We further investigated the impacts of simulated phosphorylation on anchorage independent tumor spheroid growth. Since the outcomes of this correlate assay tended to have greater clonal variability than other assays herein, we evaluated and presented the data using spheroid diameters to compare relative fractions of medium and large spheroid growth separately. Both UGDH WT and S316D expressing lines yielded 2–3-fold greater numbers of medium spheroids (Fig. 7C) and 3–4-fold more large spheroids (Fig. 7D) in anchorage independent culture conditions, relative to cells expressing the VC and UGDH S316A constructs. Total numbers of tumor spheroids were ≈25 % or 80 % higher for UGDH S316D lines 1 or 2 respectively, relative to UGDH WT (OE). Spheroid growth was strongly reduced by treatment with RSK2 inhibitor, which almost completely suppressed medium and large spheroid growth of VC and UGDH S316A cell lines (Fig. 7E). Although spheroid growth by UGDH WT (OE) and S316D expressing cells was diminished by ≈80 % in the presence of the RSK2 inhibitor, some increased resistance was noted as these lines all continued to produce ≈10-fold more spheroids relative to the VC line (Fig. 7E). UGDH WT (OE) and S316D expressing cells were sensitive to enz suppression of large spheroid growth but nonetheless retained ≈3-fold greater resistance to enz treatment relative to the VC line (Fig. 7F). Overall, these results are consistent with significant functional consequences of UGDH S316 phosphorylation on increased production of glycans and consequent tumorigenic phenotype of prostate tumor cells, as well as resistance to pharmacological therapeutic agents.

**Fig. 8.**
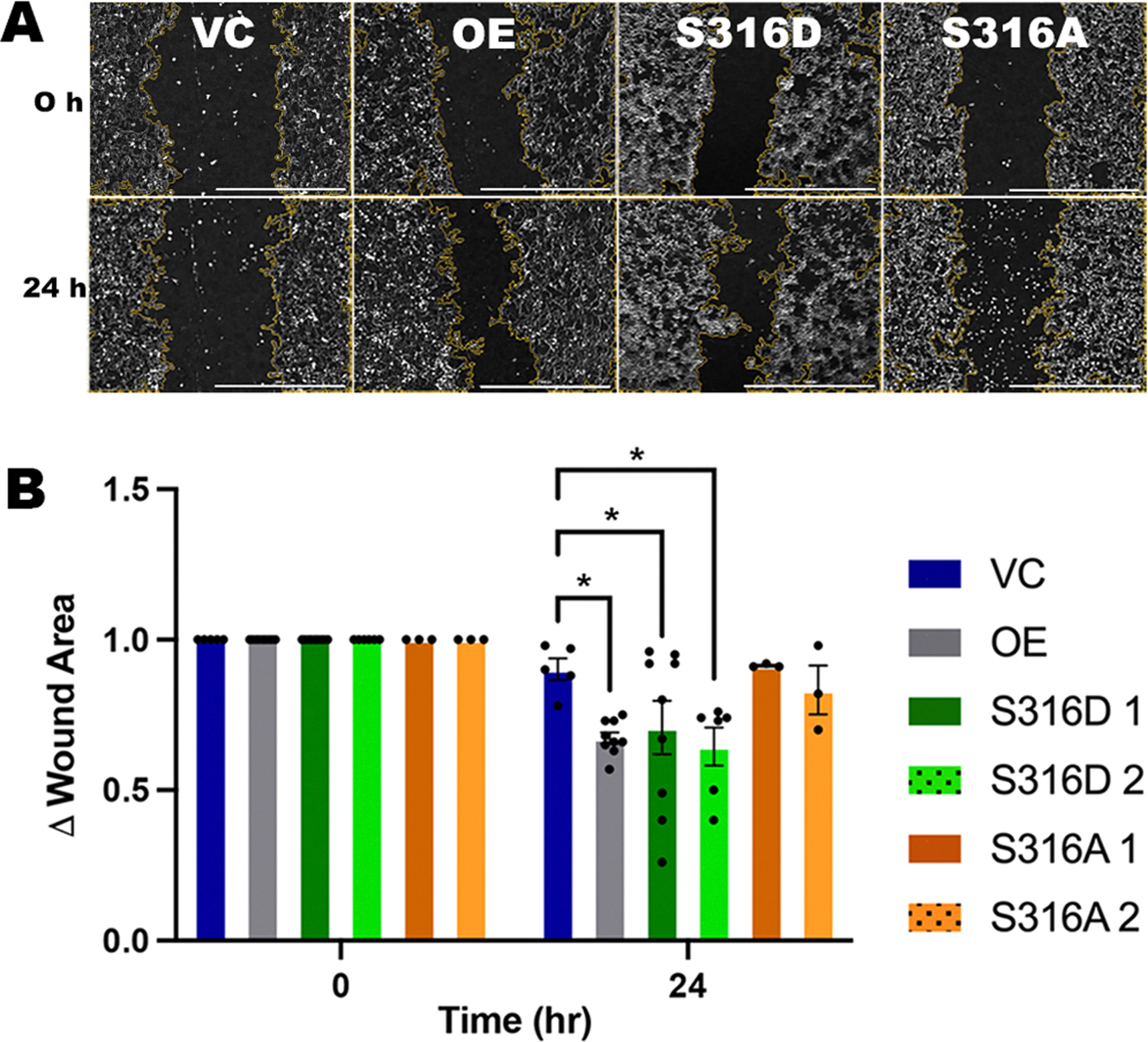
Phosphomimetic UGDH expression significantly increases cell motility. Representative images of the indicated lines in culture at time 0 and 24 h following scratch wounding of the monolayer culture are shown with object masks to indicate area in which wound closure was quantified (A). Scale bars indicate 1000 μm. (B) Wound area digitally measured at 24 h was normalized to the area at time 0 for the identical coordinates. Wound closure is plotted as the fraction of original wound area for each indicated cell line as the mean ± SEM for at least three sites per scratch in triplicate cultures, repeated three times. Individual data points are shown; * *p* < 0.01.

Finally, the excess presence of cell surface and extracellular GAGs is a hallmark of numerous tumor cell types that contributes to both accelerated growth and enhanced motility [41–45] so we compared motility among the cells using a wound closure assay in cell monolayer cultures. Cells were pre-treated with the anti-proliferative agent mitomycin C, to ensure that the enhanced proliferation rates of UGDH WT (OE) and S316D cell lines did not impact the outcome. Significantly higher intrinsic motility of UGDH WT (OE) and S316D expressing cells was observed by the ability of these lines to reduce the wound area 30–35 % by 24 h after wound induction (Fig. 8A and Fig. 8B). In contrast, VC and UGDH S316A cell lines displayed minimal wound area reduction, as we have previously observed for the parental LNCaP cell line, which is poorly motile [46]. Collectively, the results presented here support a model in which the overexpression of UGDH promotes pro-tumorigenic glycan production and suppresses androgen elimination, which is dependent on phosphorylation of UGDH S316 downstream of AGC kinase activation by EGF and DHT, and is manifested in therapeutic resistance, anchorage independent growth, and enhanced cellular motility.

## Discussion

**Fig. 9.**
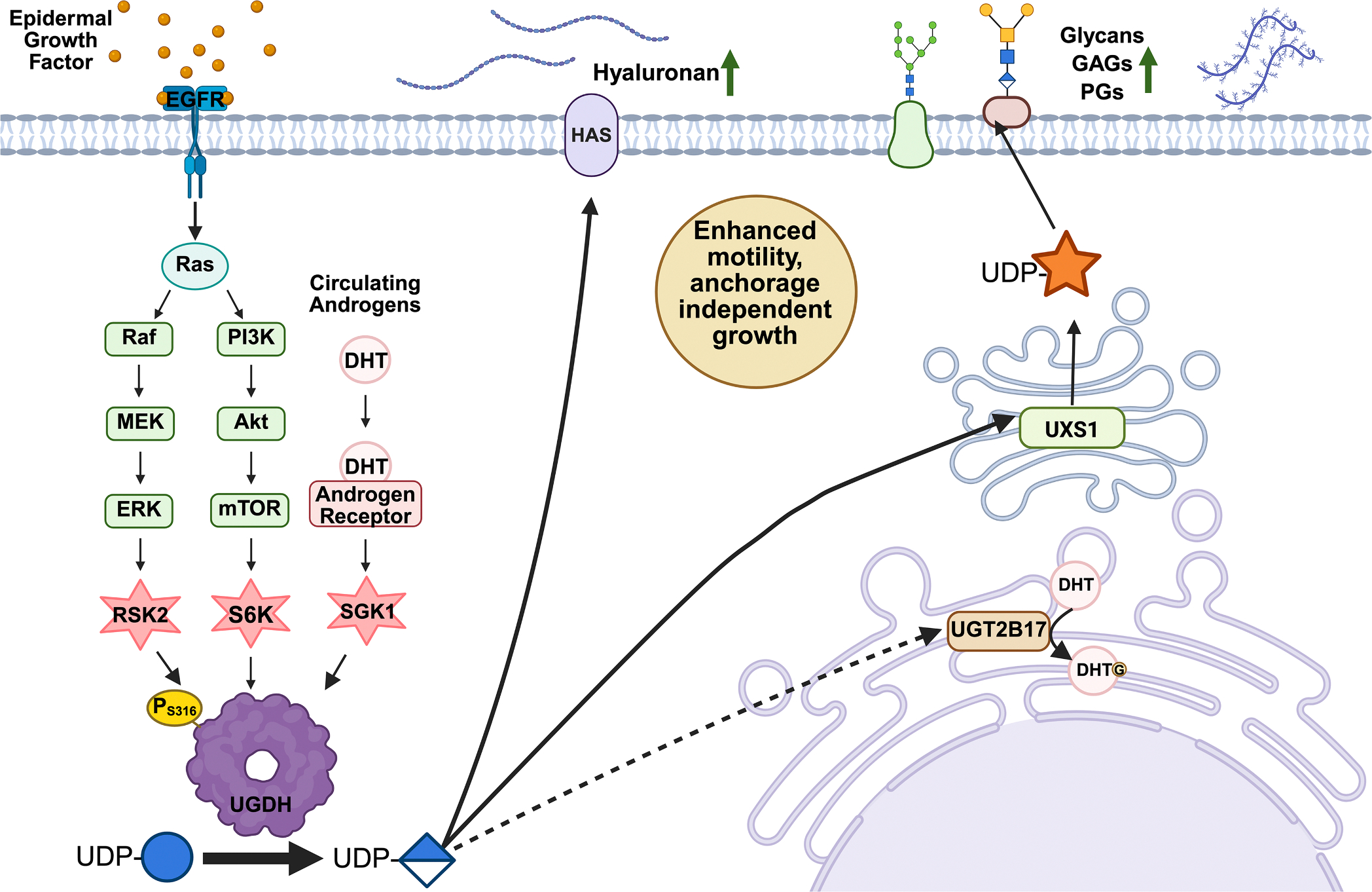
Model for the mechanism by which UGDH acts as a functional sensor for UDP-GlcA partitioning among critical cellular fates. Extracellular factors such as androgens and growth factors trigger signaling cascades through their respective effector pathways, ultimately activating RSK2, S6K1, and SGK1 to phosphorylate UGDH S316. Resulting increased activity of UGDH directs greater flux of its product, UDP-GlcA, to the cell surface for HA synthesis and secretion, as well as to the Golgi apparatus to support proteoglycan production and to the cytosolic face of the ER for glycan synthesis. The model predicts these functions occur in a manner that significantly reduces availability of UDP-GlcA for UGT2B17-catalyzed glucuronidation of androgens in the ER lumen. Generic cartoon shapes are used to represent hypothetical proteins and proteoglycans, and are not intended to represent specific structures. HAS, hyaluronan synthase; GAG, glycosaminoglycan; PG, proteoglycan; DHT-G, DHT-glucuronide. Symbols for glucose (blue circle), GlcA (blue and white diamond), GlcNAc (blue square), and Xyl (orange star) are consistent with Symbol Nomenclature for Glycans. Created in https://BioRender.com.

Understanding the basic mechanisms underlying metabolite use in critical pathways that modulate cancer initiation and progression is essential to improve response to therapies such as androgen deprivation, where castration resistance is promoted by UDP-GlcA being diverted away from glucuronidation. It is also important to improve our knowledge of cellular sensing components that coordinate extracellular signals to direct intracellular metabolites into macromolecular biosynthesis. Many multi-subunit enzymes have been well characterized as coordination points for nutrient and growth factor cues, such as AMP-activated kinase and mTORC1 [47,48]. In this study, we determined that UGDH, which is the sole enzyme responsible for UDP-GlcA partitioning to the compartmentalized processes that segregate glycan biosynthesis from androgen glucuronidation, is phosphorylated in LNCaP cells at a conserved AGC kinase consensus sequence containing S316 within the UGDH dimer association interface. Three kinases implicated in prostate tumor cell proliferation, motility, and castration resistance, RSK2, S6K1, and SGK1, mediate UGDH phosphorylation in a manner that is pharmacologically sensitive and results in pro-proliferative glycan synthesis. Moreover, simulated phosphorylation at this residue demonstrates the significant impact of p-UGDH on UDP-GlcA partitioning to glycan biosynthesis at the expense of androgen glucuronidation despite the endogenous regulatory control of UDP-Xyl, and the resulting amplification of tumor-promoting processes such as growth and motility (Fig. 9). Thus, UGDH is emerging as a new sensory switch between use of UDP-GlcA for glycan synthesis and use of UDP-GlcA for androgen (and other lipophile) elimination.

The elevation of oncogenic proteoglycans and GAGs on tumor cells is well documented in both solid and circulating cancer types [41–45]. In this study, we found that the phosphorylation of UGDH may be a mechanism by which cellular nucleotide sugar flux could be redirected to glycan output. When UGDH WT is overexpressed, whole cell UGDH activity and levels of the UDP-GlcA and UDP-Xyl products are increased in a kinase-dependent manner, which reflects the larger pool of enzyme available to catalyze their formation. This is also true of UGDH S316D, which cannot be phosphorylated at S316 but simulates the larger pool of p-UGDH. The reduced effects of kinase inhibitors on UGDH S316D cell growth supports this model, since they only impact phosphorylation of the endogenous UGDH pool. Response to 4-MU is also consistent with the model, since it shows that UGDH WT is still active and operating at a production threshold that can be scavenged, whereas UGDH S316D is continuing to produce O-glycans so the UDP-GlcA levels are not appreciably depleted by scavenging.

The elevated expression of UXS1 in conjunction with UGDH WT and S316D overexpression is significant, since it has been found that the targeted knockdown of UXS1 in tumor cells with high UGDH expression eliminates the UDP-Xyl mediated suppression of UGDH activity and leads to a toxic buildup of UDP-GlcA, disrupting organelle morphology and function [49]. The elevated presence of p-UGDH upon continued aberrant AGC kinase activation may be part of the signal for UXS1 elevation. UGDH S316A expression did not contribute to the increased expression of UXS1, nor to the p-UGDH pool and accordingly did not increase levels of glycans, sGAGs, or HA. Importantly, these lines also did not grow quickly, were not migratory, and retained higher sensitivity to growth inhibition by all three kinase inhibitors and enz.

The increased production of glycans, sGAGs, and HA by UGDH WT and S316D is particularly interesting when considered in light of UDP-Xyl binding, which was 2-fold lower in UGDH S316D protein. In the cell, UDP-Xyl concentration is normally very low so its competitive inhibition of UGDH is one of the mechanisms for preserving balance among UDP-sugar concentrations to regulate stoichiometry of their incorporation into growing proteoglycan attachments. Phosphorylated UGDH could be expected to also have reduced affinity for UDP-Xyl, which would lead to UGDH producing more UDP-GlcA, necessitating the increased expression of UXS1 and of proteoglycans and HA, to continue relieving the toxicity of UDP-GlcA. However, because UGDH S316D expression also reduces the levels of UGT2B17, the net outcome ultimately favors biosynthesis at the expense of glucuronidation.

Overall, despite the ≈2-fold reduction in V_max_ of UGDH S316D *in vitro* (Table 1), cellular levels of UDP-Glc and UDP-GlcA were not different between the UGDH WT (OE) and UGDH S316D cell lines (Fig. 4), suggesting the S316D mutation has minimal impact on the intrinsic enzymatic activity in the cellular context. The data *in vitro* also show modestly increased conformational flexibility specifically in the UGDH S316D, possibly within elements of secondary and tertiary structure that support the dimer interface, and/or lower energy non-covalent interactions between the dimers. It is tempting to speculate that the phosphorylation of UGDH at S316 would cause a minimal or transient disruption of the dimer-dimer interface. This could expose a novel surface for UGDH to interact with other cellular regulators and/or other compartmentally specific proteins in a phosphorylation-dependent manner. It is important to note that our studies with UGDH S316D in cells would be expected to yield a disproportionate number of “p-UGDH” subunits in the hexameric enzyme. In the endogenous phosphorylated form, it is more likely that only a fraction of all UGDH subunits would contain a phosphoserine and potentially only one subunit per hexamer would be conformationally possible at a given time. However, these results are informative in that they likely represent an extreme consequence of constitutively elevated p-UGDH.

The three kinases responsible for UGDH S316 phosphorylation lie within distinct signaling cascades. RSK2 acts in the MAPK cascade, and has been previously implicated in prostate cancer [50,51]. S6K1 is downstream of the PI3K/Akt signaling axis, and has also been correlated with prostate cancer progression [30,52,53]. These kinases respond to a variety of growth factors and signaling molecules, and are both situated downstream of the Ras oncogene. Dysregulated activation of kinases within these pathways is a known feature of castration resistance, and aberrant RSK2/S6K1 phosphorylation of UGDH S316 in prostate tumor cells would be expected to promote a more proliferative, invasive phenotype, as we have demonstrated here in cellular models. SGK1, while typically responsive to cellular stress, is also positively regulated via the presence of androgens and enhances prostate cancer cell viability [54,55]. We observed increases in glycan synthesis and spheroid growth in UGDH S316D cells, which have lost their dependence on exogenous androgen addition for their proliferation and show strong resistance to anti-androgen treatment (Fig. 7), suggesting that UGDH phosphorylation promotes growth irrespective of androgen levels. However, the native phosphorylation of UGDH S316 may be partially dependent upon androgen availability, as evidenced by the increased impacts of SGK1 inhibition following steroid starvation, which are likely to be mediated through the endogenous WT UGDH present in all the lines.

Our results implicate each of three kinases in UGDH S316 phosphorylation, though it is likely the primary role of a given kinase is dictated by both prevailing extracellular and intracellular conditions. Nonetheless, the crosstalk among the three pathways allows for cells to compensate for inhibition of one of the three kinases. Combined kinase inhibition was more effective at reducing cell number than enz treatment alone even in UGDH WT and S316D cells, and the UGDH S316A lines were highly sensitive even to combined treatment with enz and a single kinase inhibitor. Our results support UGDH phosphorylation as a point of regulation for UDP-GlcA elevation and a driver of biosynthetic processes that engage HA and sGAG production to avert UDP-GlcA toxicity and thereby enable a more aggressive phenotype. Understanding the mechanisms through which UGDH drives prostate cancer progression will facilitate effective targeting of UGDH as a therapeutic intervention in prostate cancer.

## Experimental procedures

### Reagents and Antibodies.

Reagents were purchased from Thermo Fisher Scientific, Inc (Waltham, MA, USA) except as indicated below. Antibodies were purchased and diluted as follows: mouse monoclonal anti-human β-tubulin (Millipore Sigma, St. Louis, MO, USA, 1:750,000); IRDye 680 conjugated goat anti-mouse IgG (LI-COR Biosciences, Lincoln, NE, USA, 1:5000); IRDye 800 conjugated anti-rabbit IgG (Rockland, Gilbertsville, PA, USA, 1:5000); rabbit monoclonal anti-human Notch1 (D1E11), rabbit monoclonal anti-pAkt substrate (RXRXXpS*/T*), rabbit monoclonal anti-RSK2, anti-pRSK2, anti-p70S6K, anti-phospho-p70S6K, anti-SGK1, and anti-pSGK1 (Cell Signaling Technology, Danvers, MA, USA, 1:1000 for each); mouse monoclonal anti-Flag M2 and mouse monoclonal anti-GAPDH (Millipore Sigma, St. Louis, MO, USA, 1:1000 for each). Polyclonal rabbit anti-human UGDH was raised against recombinant UGDH protein purified from *E.coli* and was previously characterized by our laboratory [7]. UDP-Xylose was purchased from the University of Georgia’s Carbo-Source Services (Athens, GA, USA). UDP-Glucose and UDP-Glucuronate, β-NAD^+^, and β-NADH were acquired from Sigma Aldrich (St. Louis, MO, USA). Sequencing grade chymotrypsin was from Promega Corporation (Madison, WI, USA). Clickable γ-[(6-Azidohexyl)-imido]-ATP was from Jena Bioscience (Jena, Germany). Resazurin cell viability assay kit was from Biotium (Hayward, CA, USA). DHT (5α-Androstan-17β-ol-3-one) was purchased from Steraloids, Inc (Newport, RI, USA). Enzalutamide was from APExBIO (Houston, TX, USA). Kinase inhibitors for RSK2 (BIX02565 [29]), S6K1 (PF4708671 [30]), and SGK1 (GSK650394) were all purchased from MedChemExpress (Monmouth Junction, NC, USA).

### In vitro *kinase screen*.

Sequence alignment of the UGDH S316 peptide and predicted kinases recognizing this motif was performed using the PhosphoSitePlus database [23]. This prediction was cross referenced with tissue expression data from the literature to compile a list of the top kinases likely responsible for phosphorylating UGDH S316. Purified human UGDH protein was incubated with each purified human kinase individually in the presence and absence of 200 μM ATP for two hours at 30 °C. Resulting solutions were then subjected to filter-aided sample preparation. Briefly, proteins were treated with 5 mM DTT and incubated at 56 °C for 30 min. Proteins were denatured by addition of 8 M urea before treatment with 1.5 mM iodoacetamide. The alkylation reaction was allowed to incubate in the dark for 1 h. Samples were moved to a molecular weight cutoff filter before being washed again with 8 M Urea to remove any additional detergent. Proteins were then digested 50:1 with chymotrypsin overnight. Following digestion, peptides were quenched with 1 % formic acid and dried in a vacuum concentrator. Resulting peptides were suspended in 5 % acetonitrile with 0.1 % formic acid and analyzed on a nanoflow EASY-nLC 1200 system (Thermo Fisher Scientific) coupled to an Orbitrap Eclipse tribrid mass spectrometer (Thermo Fisher Scientific). Peptides were separated on an Acclaim PepMap 100 C18 trap column (75 μm × 2 cm, 3 μm) and an EASY-Spray C18 analytical column (75 μm × 25 cm, 2 μm, 100 Å) using a linear gradient. The gradient was established using Mobile phase A, consisting of water and acetonitrile (8:2, v/v) with 0.1 % formic acid, and Mobile phase B consisting of acetonitrile with 0.1 % formic acid, applied in the following amounts: 0 min, 5 % B; 2 min, 5 % B; 110 min, 5–40 %; 5 min, 40–95 %, 5 min, 95 %; 3 min 95–5 %. The flow rate was 300 nl/min. The parameters for Orbitrap Eclipse were as listed: ion transfer tube temperature, 275 °C; spray voltage, 2000 kV for positive mode; RF-lens ( %), 40; resolution was set at 120,000 (at *m/z* 200) for MS1 scan and 15,000 for MS2 scan; scan range, 375 to 1500 (*m/z*). Automatic maximum injection time (max IT) and automatic gain control (AGC) were used. MS/MS scan was acquired using DDA mode and HCD collision energy was set at 30 %. The data were analyzed using Proteome Discoverer Version 2.4 with SequestHT and IMP-ptmRS algorithms.

### Validation of kinase activity.

Recombinant UGDH (100 μg) was incubated for 2 h at 30 °C with 1 μg of each kinase and either activated by addition of ATP (200 μM) or a conjugatable ATP analogue, γ-[(6-Azidohexyl)-imido]-ATP. Phosphorylated UGDH was detected by western analysis (using anti-pAkT substrate antibody) or by SDS-PAGE following reaction with tetramethylrhodamine (TAMRA) alkyne, copper, THPTA, and sodium ascorbate.

### Generation and purification of recombinant human UGDH S316 point mutants.

The coding sequence for human WT UGDH was codon optimized for expression in *E. coli* then inserted into the pET28a vector. Mutagenesis primers for S316A and S316D UGDH were synthesized by Integrated DNA Technologies (IDT). Primer sequences were as follows:

S316A forward CTATCAATAATGCGGGCTGCAAAACG,

S316A reverse GATTACCAGCGTCGTCGTTTTGCAGCC,

S316D forward, CTATCAATAATGCGATCTGCAAAACGA,

S316D reverse GATTACCAGCGTCGTCGTTTTGCAGAT.

Site directed mutagenesis was done using KOD Hot Start according to the vendor’s instructions (Sigma). The plasmids were sequenced by Eton BioScience to verify the mutation and confirm integrity of the remaining sequence. For protein purification, plasmids were used to transform *E. coli* BL21(DE3) cells. Overnight colonies were picked and amplified in 2xYT medium containing 50 mg/L kanamycin at 37 °C until reaching an OD600 of 0.6–0.8. Protein expression was induced with the addition of 0.5 mM isopropyl β- d-1-thiogalactopyranoside (IPTG) and the incubation temperature reduced to 30 °C for 4 h. The cells were pelleted by centrifugation and lysed by sonication. HisTrap FF column (GE Healthcare 17-5255-01) was used to purify the protein by nickel affinity chromatography. Purified protein was concentrated to a range of 3.17 mg/mL to 8.62 mg/mL and dialyzed against 20 mM Tris pH 7.4 with 1 mM DTT overnight. Protein concentration was quantified by the absorbance at 280 nm on a Cary60 UV/vis spectrophotometer and protein was stored at −80 °C until use.

### Characterization of purified UGDH S316D and UGDH S316A.

Purified S316 mutants were compared to the UGDH WT enzyme using the following approaches as previously described [15,21,34]. Enzymatic activity was evaluated by steady state kinetics in the presence and absence of the endogenous inhibitor UDP-Xylose to compare Michaelis constants and IC_50_ values. Intrinsic stability was assessed by thermal denaturation and quaternary structure was determined by size exclusion chromatography. Binding of UDP-Xylose to recombinant UGDH and mutants was measured by isothermal titration calorimetry using the MicroCal PEAQ-ITC (Malvern Panalytical). UGDH WT (50 μM in 0.1 M sodium phosphate buffer, pH 7.4) was placed in the cell, with UDP-Xyl (250 μM) in the syringe. Binding heats were corrected for the heat of dilution and analyzed using the MicroCal PEAQ-ITC Analysis Software, employing a single-site binding model to determine K_d_ values.

### Cell culture and stable UGDH mutant expression.

The LNCaP androgen dependent human prostate adenocarcinoma cell line was purchased from American Type Culture Collection (Manassas, VA, USA). Prior to experiments, LNCaP lines were maintained in RPMI-1640 supplemented with 10 % FBS and 2 mM l-glutamine as recommended by the vendor. Primers to create S316D and S316A mutations were designed specifically for the human UGDH expression sequence (derived from UGDH EX-Q0483-Lv102, GeneCopoeia, Rockville, MD, USA), which encodes UGDH with an N-terminal Flag epitope tag. Following mutagenesis, plasmids were sequenced to confirm presence of the mutations and integrity of the remaining UGDH sequence. The point mutants were clonally selected in RPMI-1640 with 10 % FBS and 0.25–0.5 μg/mL puromycin (Gibco) and continuously maintained in this medium. LNCaP cell lines overexpressing WT UGDH or its vector control were previously generated and characterized [16]. LNCaP lines stably expressing UGDH S316A or UGDH S316D were analyzed by Western blot to confirm expression of UGDH and the Flag epitope. Six clones from each group were selected and characterized for basal gene expression. Two clones of S316A and S316D representing an average level of UGDH expression were chosen for further analysis. Cells were maintained for a maximum of 10 passages from thaw. All cell lines used in this study were authenticated by the ATCC using STR profiling.

### Cell treatments.

Cell treatments were conducted as follows, except where described. To stimulate phosphorylation of UGDH at S316 for Western blot analysis, cells were cultured in replete medium until they reached 70 % confluence. Seventy-two hours prior to harvest, medium was replaced with phenol-red-free media containing 5 % CS-FBS to reduce androgen signaling. Twenty-four hours prior to harvest, medium was replaced with phenol-red-free media containing 10 % CS-FBS plus 10 nM DHT or vehicle control. EGF (20ng/mL) or vehicle control were added to cell media twenty minutes prior to harvest. For assessment of UGDH pS316 in these conditions with kinase inhibition, assays were repeated with kinase inhibitors added alongside EGF, DHT, and controls. Kinase inhibitor treatment concentrations were optimized based on published efficacy ranges [56–58] and the following were used for all assays: BIX02565 (20 μM), PF4708671 (20 μM), GSK650394 (40 μM). Proliferation assays were performed with 10 μM enzalutamide (enz) added as described below, and 3D assays were performed with 1 μM enz added during seeding as described. For click chemistry quantification of glycans in cells treated with 4-MU, 10 μM was added to cell medium during the addition of the click labeling reagents.

### Western analysis.

Media were aspirated from cells, and a portion kept for analysis of HA content. Cells were washed with 2 mL cold 1x PBS, scraped into 1 mL of cold 1xPBS, pelleted by centrifugation, and lysed with 1x RIPA buffer supplemented with 1x protease inhibitor cocktail. For analysis of phosphoprotein expression, 1x phosphatase inhibitor was added to the lysis buffer. Bradford assay was performed to quantify protein content of cell lysates. Blots were imaged and quantified through fluorescence emission using the Odyssey Infrared Imaging System (LI-COR Biosciences, Lincoln, NE, USA). β-tubulin or GAPDH were used to normalize protein expression. Each analysis was plotted for three technical replicates of a representative experiment that was repeated at least three times. Statistical significance was assessed by one-way ANOVA followed by Tukey’s post hoc test for multiple comparisons.

### Specific activity of UGDH in whole cell lysates.

Stable LNCaP cell lines overexpressing UGDH WT and S316 mutants were assayed for UGDH-specific activity as previously described [34] Briefly, cells were cultured in 10 cm plates, washed three times with cold 1xPBS, and centrifuged at 1500×*g* for 5 min. Cells were resuspended in twice the pellet volume of lysis buffer (50 mM Tris–HCl pH 7.4, 150 mM NaCl, 1 mM EDTA, with a protease inhibitor cocktail). Samples were transferred to tubes with an equal volume of acid washed glass beads (Sigma), placed in cryo blocks chilled to −80 °C, and lysed in the Geno/Grinder (Spex) at 1500 rpm for 5 cycles of 45s/15 s rest. The resulting supernatant was chilled on ice for 1 hr and centrifuged at 13,000 rpm for 15 min to obtain final lysates. Enzymatic activity of the lysates (100 μg) was assayed with 0.5 mM UDP-glucose and 1 mM NAD^+^ in the presence or absence of 1 mM UDP-Xyl, and monitored for changes in NADH (Abs 340 nm). Reaction rates for samples containing UDP-Xyl were subtracted from samples without UDP-Xyl to obtain UGDH-specific activity reported as [NADH] in nmol/min/mg lysate. Each cell line analyzed contained three or more technical replicates for reactions with and without UDP-Xyl plotted as mean ± SEM. Statistical significance was assessed by one-way ANOVA (Prism).

### Quantification of UDP-sugars.

Lysates from the indicated lines and treatments were extracted with butanol-saturated concentrated formic acid and analyzed for UDP-sugar content by liquid chromatography and mass spectrometry (LC-MS) according to published methods [5,6,59, 60]. Statistical significance was assessed by one-way ANOVA followed by Tukey’s post hoc test for multiple comparisons.

### Glycan analysis.

To compare differences in glycan levels between WT UGDH, UGDH S316A, UGDH S316D, or vector control, 2.0 × 10^4^ cells of each type were seeded into 6-well plates in complete medium containing 50 μM tetraacetylated N-azidoacetylgalactosamine (Ac_4_ GalNaz) or tetraacetylated N-azidoacetylglucosamine (Ac_4_GlcNaz) (both from VectorLaboratories, Newark, CA, USA) or DMSO control. Cells were grown for 72 h and then lysed as described above. Equal protein from each lysate was subjected to the cyclooctyne click chemistry conjugation via reaction with tetramethylrhodamine-PEG4-alkyne and tris-hydroxypropyltriazolylmethylamine ligand [35,36] (VectorLaboratories). This method was also performed for the clickable-ATP kinase screens [25]. Following organic solvent cleanup to remove excess reagents, samples were run on an SDS-PAGE gel and fluorescence at 532 nm was visualized with an iBright FL1500 Imaging System (Invitrogen). Gels were stained with GelCode Blue to normalize fluorescent signals to total protein content per sample. Analysis was performed with iBright Analysis Software (Invitrogen).

### Quantification of sulfated glycosaminoglycans and hyaluronan.

Relative quantities of sulfated glycosaminoglycans (sGAGs) were measured via the colorimetric 1,9-dimethylmethylene blue (DMMB) assay [38]. Cells were seeded at 1.5 × 10^5^ per well of a 6-well plate and cultured in phenol-red-free complete medium for 48 h. Cells were then subjected to digestion with papain (Sigma) for two hours at 60 °C to liberate GAGs from core proteins. The solubilized cell extracts were mixed with DMMB dye at pH 1.5 (Sigma) and absorbance values at 595 nm and 525 nm were measured immediately using a Cytation5 Cell Imaging Multimode reader (Agilent Technologies). Corrected sample absorbance was obtained by subtracting the 595 nm value from the 525 nm value, compared to a standard curve of chondroitin sulfate A (Biosynth, Louisville, KY) and sGAG content was extrapolated via linear regression. Wells were assayed in triplicate for each cell line and sGAG quantities were normalized to cell count determined by resazurin viability assay as described below. All data were plotted for three technical replicates of experiments that were repeated three times. Statistical significance was assessed by one-way ANOVA followed by Tukey’s post hoc test for multiple comparisons.

HA was quantified by a competitive binding assay as previously described [7,15,39]. Briefly, high molecular weight HA (Lifecore) was coupled to Covalink 96-well plates at 200 μg/ml in ddH_2_O in the presence of 25 μg Sulfo-NHS/EDC for 90 min, then washed and blocked. Serial dilutions of cell culture media were incubated with biotinylated HA binding protein (bHABP, Millipore Sigma) at a final concentration of 1 μg/mL. Standards and samples were incubated in the HA-precoated wells at room temperature overnight then washed four times with PBS/0.05 % Tween 20. The plate was developed using the Vectastain Elite ABC avidin-biotin HRP system (VectorLaboratories) with TMB (Thermo Fisher) as substrate, and absorbance was measured at 650 nm. HA concentration was interpolated from a standard curve and normalized to cell count.

### Quantification of DHT-glucuronides.

Cells were cultured in standard media in 24-well plates to 50 % confluence. For basal conditions, standard replete medium was replaced for 48 h followed by harvest of the media and determination of cell count. For DHT-stimulated conditions, standard media were removed and cells were given phenol red-free RPMI 1640 supplemented with 1 % charcoal-stripped FBS for 48 h (androgen free conditions). These media were then removed and replaced with fresh androgen free medium containing either vehicle or 10 nM DHT. Media were collected and cells were counted 48 h later. Conditioned media were stored at −80 degrees until extraction for LC/MS quantification. Conditioned media from the indicated lines and treatments were extracted using a C18 Tip (Pierce), which was first wet in 50 % acetonitrile and equilibrated with 100 μL 0.1 % trifluoroacetic acid (TFA) for each 100 μL of sample. Following a TFA rinse, the sample was eluted with 100 μL of 0.1 % formic acid in acetonitrile. Samples were vacuum dried and redissolved in 50ng/mL internal standard in methanol/water (50:50, v/v). Samples were fractionated on a Thermo Scientific Accucore C18 column (2.6 μm, 100 mm × 2.1 mm) equilibrated in Buffer A (water + 5 % acetonitrile + 0.1 % formic acid) and eluted by a linear gradient of 2 % to 98 % Buffer B (acetonitrile + 5 % water + 0.1 % formic acid). Mass spectrometry was performed using Thermo Scientific TSQ Altis triple quadrupole in negative-ion mode. Samples were quantified by interpolation on a standard curve, normalized to cell number (1 × 10^6^), and statistical significance was assessed by ANOVA followed by Tukey’s post hoc test for multiple comparisons.

### Proliferation assay.

For basal growth, 5 × 10^4^ cells of each line were cultured in 24-well plates in standard steroid-replete media. Cell proliferation was measured daily for three days in replicate plates incubated for 2 h with media containing 10 % resazurin, after which fluorescence of the reduced product, resorufin, was measured (560Ex/590Em) in a microplate reader. To assess the impacts of kinase inhibition on cell growth, assays were repeated in the presence of inhibitors individually and in combination. For androgen-mediated cell growth with kinase inhibition, cells were cultured in 24-well plates in standard steroid-replete media. After 12 h, media were first removed and replaced with media containing 5 % CS-FBS (androgen-depleted conditions) for 48 h, and then media were removed and replaced with media containing 10 % CS-FBS plus 10 nM DHT and kinase inhibitors for another 48 h. Cell proliferation was measured daily as described above beginning when cells were switched to androgen free conditions. For enz treatments, cells were cultured in 24-well plates in standard steroid-replete media. After 12 h, media were replaced with replete media containing vehicle or 10 μM enz and vehicle or kinase inhibitors. For assays with kinase inhibition, cell proliferation was measured daily as described above beginning when cells were switched to the respective treatment condition. Cell numbers were extrapolated from fluorescence values by comparison to a standard curve for each respective cell line, and cell count at the end of the experiment was normalized to cell count on day zero directly following media replacement. All data were plotted for four technical replicates of experiments that were repeated three times. Statistical significance was assessed by one-way ANOVA followed by Tukey’s post hoc test for multiple comparisons.

### Tumoroid growth assay.

For three-dimensional cell culture to compare anchorage independent growth rates of the stable cell lines selected for overexpression of WT UGDH, UGDH S316A, UGDH S316D, or vector control (VC), 2.5 × 10^4^ cells of each type were seeded in 96-well plates in phenol-red free RPMI 1640 containing 1.2 % methylcellulose, 10 % FBS, l-glutamine, and puromycin (*n* = 3). Wells were imaged on day 1, day 4, and day 7 post-seeding using a Cytation5 Cell Imaging Multimode reader (Agilent Technologies). To obtain spheroid count and size from each image, cell analysis via object masking was performed with Gen5 Data Analysis Software (Agilent). Spheroids were categorized by size as single cells (0–10 μM), small (11–49 μM), medium (50–99 μM), or large (>100 μM) spheroids. Data were plotted for combined numbers of medium and large spheroids determined in three technical replicates of experiments that were repeated three times. Statistical significance was assessed by one-way ANOVA followed by Tukey’s post hoc test for multiple comparisons.

### Motility assay.

Cell motility was assessed by modified pipette tip gap closure migration assay [61,62]. Cells were plated in replete medium at 3 × 10^5^ cells in each well of a 24-well plate to achieve a consistent monolayer. Twelve hours after plating, cells were treated with 5 μg/mL mitomycin C (Cell Signaling Technologies) for two hours to account for differences in basal proliferation rates among the cell lines. Scratches were performed manually with a sterile P10 pipette tip. Immediately following scratch formation, cell medium was carefully replaced to remove mitomycin and any cells that had detached. Bright-field images of representative sections of each scratch were taken with a Cytation5 imager at 0 h, 24 h, and 48 h following media replacement. Image parameters were set following instrument manufacturer protocols [61] and object masking was performed to obtain wound area at each time point. Final wound area was normalized to the initial wound area to determine the percent change. Three to nine representative gap areas from at least three separate wells were utilized for each cell line. Statistical significance was assessed by one-way ANOVA followed by Tukey’s post hoc test for multiple comparisons.

## Supplementary Material

1

Supplementary materials

Supplementary material associated with this article can be found, in the online version, at doi:10.1016/j.matbio.2025.10.004.

## Figures and Tables

**Table 1:** Comparison of activity and properties of purified UGDH WT and S316 mutants.

UGDH	Tm (°C)		UDP-Glucose		NAD+		UDP-Xylose	
	Apo	Holo	K_m_(μM)	V_max_*	K_m_(μM)	V_max_*	K_d_(nM)	IC_50_(nM)

S316A	49±0.2	66±3.0	36±6	550±25	380±60	490±20	71±20	250±60
S316D	47±1.0	63±0.3	19±5	310±10	330±70	230±20	150±30	250±30
WT	50±0.4	68±1.7	12±3	640±30	530±90	710±40	91±35	220±50

* nmol/min/mg of protein.

## Data Availability

Data will be made available on request.

## Abbreviations:

UGDH: UDP-glucose 6-dehydrogenase
RSK2: Ribosomal S6 Kinase isozyme 2
S6K1: p70 S6 kinase
SGK1: Serum/glucocorticoid-regulated kinase 1
WT: wild-type
enz: enzalutamide
