# Supplementary material for: Phosphorylation of UDP-glucose dehydrogenase increases glycosaminoglycan biosynthesis and promotes tumor cell motility, spheroid growth, and therapeutic resistance
Source: Matrix Biol. Author manuscript; Available in PMC 2026 Jul 3. (PMC13330650; doi:10.1016/j.matbio.2025.10.004)
Supplement: 1 [file NIHMS2184803-supplement-1.docx]

**Supporting information**

**Phosphorylation of UDP-glucose dehydrogenase increases glycosaminoglycan biosynthesis and promotes tumor cell motility, spheroid growth, and therapeutic resistance**

Asher R. Utz^1^, Linlin Ma^1^, Dalton Hilovsky^1^, Brenna M. Zimmer^1^, Emily Allego^1^, Jade Fluharty^1^, Pooja Narasimhan^1^, Jeffrey R. Enders^2^, George Grady^1^, Monica Milici^1^, Pengda Liu^3^, Xiaojing Liu^1^, Joseph J. Barycki^1^, and Melanie A. Simpson ^1^*

^1^ Department of Molecular and Structural Biochemistry, North Carolina State University

^2^ Molecular Education, Technology and Research Innovation Center, North Carolina State University

^3^ Department of Biochemistry and Biophysics, University of North Carolina - Chapel Hill

*To whom correspondence should be addressed: Department of Molecular and Structural Biochemistry, North Carolina State University, 120 Broughton Drive, Raleigh, NC 27695-7622; Tel (919) 515-5680; E-mail: msimpso3@ncsu.edu

**List of material included:**

**Supplementary Figures and legends**

**Supplementary Figure 1**

**Supplementary Figure 2**

**Supplementary Figure 3**

**Supplementary Figure 4**

**Supplementary Figure 5**

**Supplementary Figure 6**

SFigure 1.


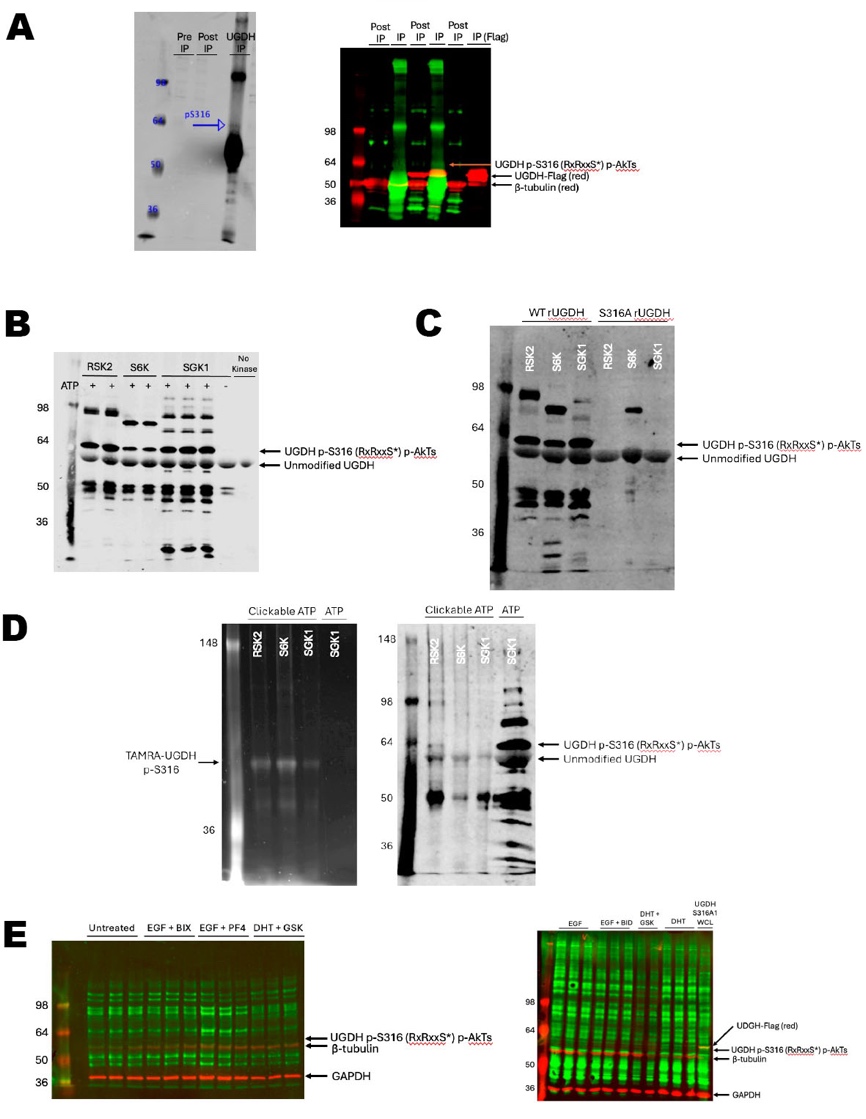


**Supplementary figure 1**. Supporting evidence for Figure 3 of the manuscript. A) Whole cell lysates of LNCaP cells were immunoprecipitated with anti-UGDH or anti-Flag antibody and immunoblotted for UGDH and p-Akt substrate (p-AktS). Arrows indicate the relevant bands for endogenous UGDH and Flag epitope-tagged transgenic UGDH. Pre- and post-IP lysates were included alongside the IP sample. Three biological replicates are shown. The strong band at ≈50kDa in each image corresponds to the heavy chain IgG in the immunoprecipitate. B) Full western blot image for Figure 3A. C) Full western blot image for figure 3B. D) Full fluorescent images for figure 3C. E) Western blot images supporting densitometric results presented in figure 3D.

SFigure 2.


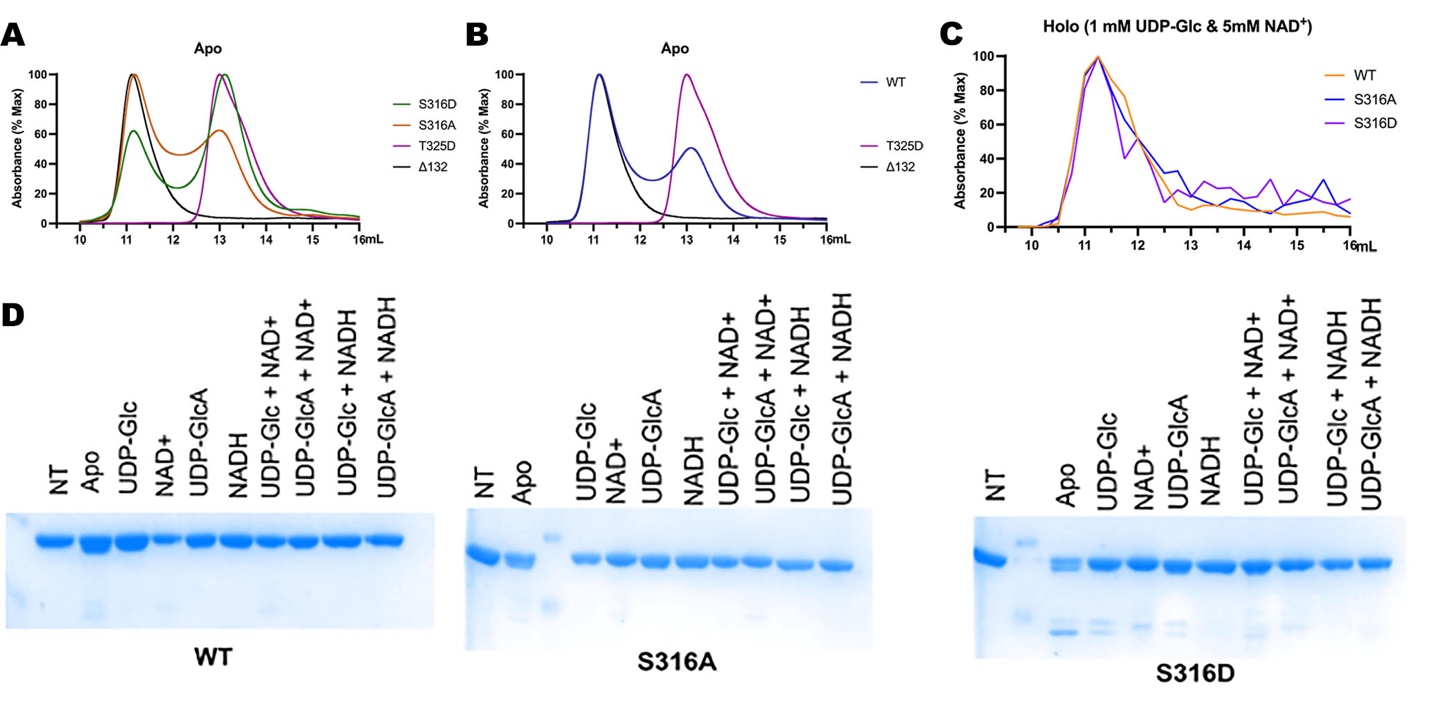


**Supplementary Figure 2.** UGDH S316D and S316A mutants exhibit comparable in vitro properties to WT UGDH. Purified WT and mutant UGDH were separated by gel filtration chromatography to assess impacts on quaternary structure. In apo conditions (A), UGDH S316D and S316A mutants elute in peaks corresponding to the dimeric and hexameric states adopted by UGDH T325D and UGDH Δ132, respectively, and previously validated by SEC. The elution traces for these two UGDH species were collected alongside the UGDH S316 mutants to confirm the elution profiles. (B) The elution profile for the wild-type apoenzyme is shown superimposed on those of the dimeric and hexameric control species for comparison. (C) In the presence of 1mM UDP-Glc and 5mM NAD^+^, both UGDH S316D and S316A mutants were fully stabilized to the hexamer conformation. (D) UGDH S316D displayed modestly increased susceptibility to limited trypsin digestion in apo and holo conditions compared to UGDH WT or UGDH S316A. Results are representative of at least three different experimental replicates. NT, no trypsin control.

SFigure 3.


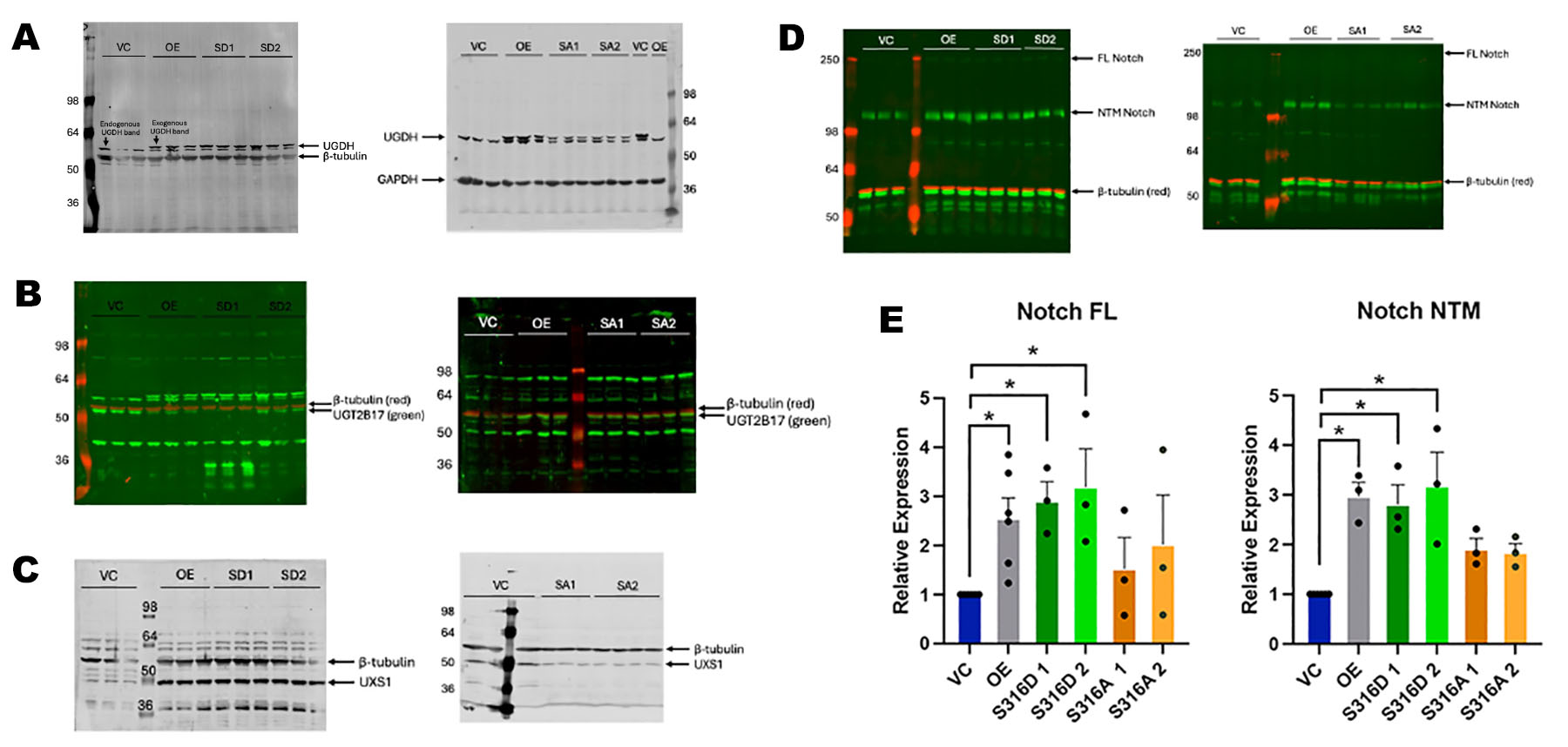


**Supplementary Figure 3.** Supporting blots are shown for densitometric quantification presented and analyzed in Figure 5 of the manuscript. A) UGDH expression is indicated as endogenous UGDH and Flag epitope-tagged UGDH to distinguish the transgenes in the UGDH wild-type overexpression line (OE) relative to UGDH S316D (SD1 and SD2, two independently selected lines) and S316A (SA1 and SA2 independently selected lines) clonal variants. Additional blots are shown for UGT2B17 (B), UXS1 (C), and Notch1 (D). Notch1 quantification with individual points and significance are shown in (E) to complement Figure 5.

S Figure 4.


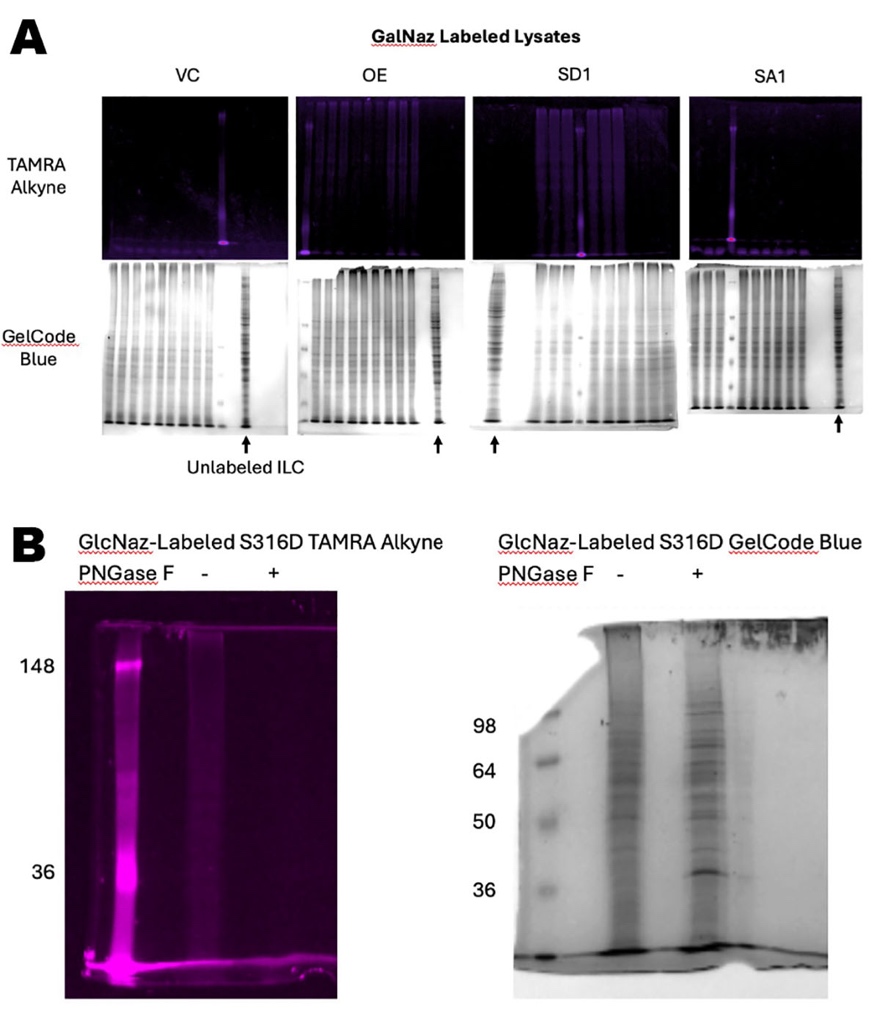


**Supplementary figure 4**. Representative images presented support figures 6A and 6B of the manuscript. LNCaP stable cell lines as indicated were metabolically labeled with either GalNAz (to detect synthesis of O-glycans quantified in Figure 6A) or GlcNAz (to detect N-glycans). A) Upper panels show images of PAGE gels run with multiple replicates of each cell line. Lower panels show total protein in the same gels, stained with GelCode Blue. B) N-glycans were measured by tagging with GlcNAz and the dependence of the fluorescence signal on N-glycan incorporation was confirmed by pretreatment of samples with protein N-glycosylase (PNGaseF) prior to electrophoresis (left). The right panel shows total protein.

S Figure 5.


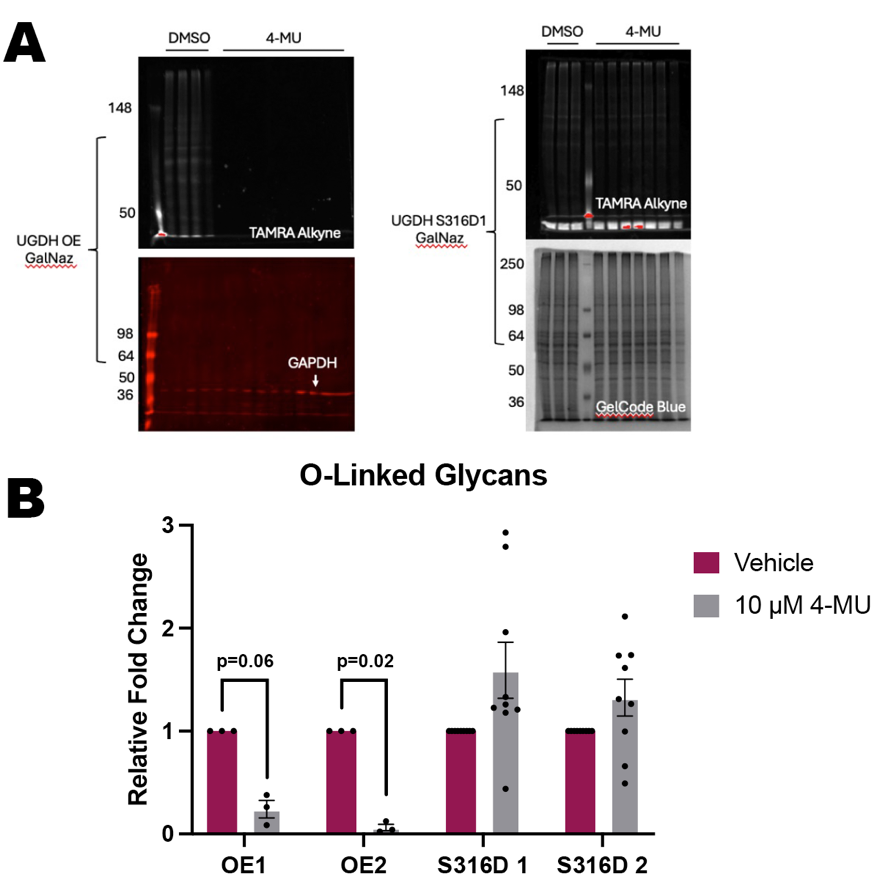


**Supplementary figure 5.** UGDH S316D expressing cells uniquely resist suppression of glycans by 4-MU treatment. A) UGDH WT (OE) and S316D cells were treated with 4-MU and Ac_4_GalNaz labeling reagent for 72 hours prior to click conjugation and analysis via SDS-page. Equal amounts of protein from each sample as determined by Bradford assay were loaded on the gel. Fluorescence in the presence of 4-MU was normalized to vehicle (DMSO) treatment conditions. The top panels show fluorescence of labeled samples; bottom left is blotted for GAPDH as a loading control; bottom right is stained for total protein using GelCode Blue. B) Images were digitally quantified for each condition and results were plotted relative to the vehicle-treated UGDH OE control. UGDH OE cells showed significant reductions in O-linked glycans, but this was not observed in S316D mutant lines. Results represent three experimental replicates. Mean ± SEM is plotted with individual data points shown. Statistical significance is indicated.

SFigure 6.

**Supplementary Figure 6.** Combined kinase inhibition is more effective at reducing cell number than enz alone. Cells were treated in the presence or absence of indicated inhibitors and enz for three days and cell numbers were determined by measuring the conversion of resazurin to resorufin as described in Experimental Procedures. Cells treated with all three kinase inhibitors in tandem showed the largest reduction in cell number, exceeding that of the enzalutamide only treatment group. Mean ± SEM of three independent experiments is plotted with individual data points shown. All treatment results were statistically significant (p<0.01) relative to the untreated control for each line, except for the enz treatment alone in the UGDH WT and S316D lines, where there was minimal detectable effect on proliferation.
